# Microbial and metabolic profiles associated with HPV infection and cervical intraepithelial neoplasia: a multi-omics study

**DOI:** 10.1128/spectrum.00192-25

**Published:** 2025-04-30

**Authors:** Xiaowen Pu, Xiao Wang, Jingjing Wang, Zhengrong Gu, Haiyan Zhu, Chao Li

**Affiliations:** 1 Department of Gynecology, Shanghai First Maternity and Infant Hospital, School of Medicine, Tongji University, Shanghai, China; 2 Shanghai Key Laboratory of Maternal Fetal Medicine, Shanghai Institute of Maternal-Fetal Medicine and Gynecologic Oncology, Clinical and Translational Research Center, Shanghai First Maternity and Infant Hospital, School of Medicine, Tongji University, Shanghai, China; University of Nebraska-Lincoln, Lincoln, Nebraska, USA

**Keywords:** cervical intraepithelial neoplasia, microbiome, metabolome, HPV

## Abstract

Cervical cancer is the most common malignancy of the female reproductive system, with the incidence of human papillomavirus (HPV) being a crucial factor in its pathogenesis. Emerging evidence indicates that cervicovaginal microbiota may influence HPV persistence and cervical intraepithelial neoplasia (CIN). However, the interplay between cervicovaginal and cervical tissue microbiomes and their association with HPV infection and CIN remains poorly understood. In this cross-sectional study, we analyzed the microbiota profiles of cervicovaginal and cervical tissue via five-region 16S rRNA gene metabarcoding, along with cervicovaginal metabolites, including short-chain fatty acids (SCFAs) and non-targeted metabolomic data, from 94 women. Key species, particularly *Lacticaseibacillus* and various anaerobes, are vital components of the microbiota found in both cervicovaginal secretions and cervical tissue, despite notable differences in microbial composition. The CIN group exhibited significant differences in microbial diversity and composition compared to the control groups, with key species such as *Lacticaseibacillus iners* and *Prevotella bivia* associated with HPV status and CIN progression. Metabolomic analysis revealed alterations in glycerophospholipid metabolism, but not in SCFAs, with correlations observed between metabolites and HPV status. Notable associations, including *P. bivia*–PE(18:1/0:0)–HPV and *Fusobacterium periodonticum*–PI(40:6)–HPV, were identified. Our findings emphasize the critical roles of cervicovaginal and cervical tissue microbiomes in HPV infection and CIN development, highlighting specific microbial species and metabolic pathways for early detection and therapeutic targets.

**IMPORTANCE:**

Cervical cancer is the most prevalent malignancy in the female reproductive system, with human papillomavirus (HPV) persistency being a critical factor in its pathogenesis. This study highlights the significant yet often overlooked role of cervicovaginal secretion and cervical tissue microbiota in influencing HPV infection and the progression of cervical intraepithelial neoplasia (CIN). By employing a multi-omics approach, we elucidated distinct microbiota profiles in cervical tissues compared to cervicovaginal secretions, revealing a complex interplay between specific bacterial species (notably *Lacticaseibacillus* and anaerobes) and metabolomic changes associated with glycerophospholipid metabolism. Our findings address a significant gap in understanding the interplay between cervicovaginal secretion and cervical intratissue microbiomes, HPV infection, and CIN.

## INTRODUCTION

Cervical cancer is the most prevalent malignancy of the female reproductive system, with its incidence rates progressively increasing (1). In China, the age-standardized incidence rate in 2022 was 13.83 per 100,000, and the mortality rate was 4.54 per 100,000 (2). Persistent detection of human papillomavirus (HPV) in cervical cells has been identified as a critical factor for the development of cervical intraepithelial neoplasia (CIN) and cervical cancer (3). However, the factors influencing HPV persistence and the pathogenesis of CIN and cervical cancer remain incompletely understood.

Emerging evidence suggests that the cervicovaginal microbiota plays a significant role in the persistence of genital HPV infection, CIN, and even cervical cancer (4, 5). Studies have found that HPV-positive women are more likely to exhibit *Lacticaseibacillus iners*-dominated (community state type [CST] III) or low-*Lacticaseibacillus*-bacterial vaginosis (BV) states, which correlate positively with the severity of cervical lesions and specific HPV types (4, 6–13). These findings highlight that disturbances in the cervicovaginal microbiota composition can lead to a pro-inflammatory microenvironment, significantly impacting the outcomes of HPV infections (14, 15). Additionally, recent studies indicate that the microbiota can colonize tissues through mucosal sites, affecting biological processes such as inflammation and metabolism within the tissue microenvironment (16, 17), thus shedding light on the interactions between microbiota and disease development (18). Despite this, few studies have investigated the role of intratissue microbiomes in HPV infection and CIN development (19, 20), and the relationship between cervicovaginal and intratissue microbiomes remains unclear.

Vaginal metabolism reflects the characteristics of the vaginal microenvironment and host response, potentially influencing systemic health (21). Research indicates that HPV infection significantly alters the vaginal metabolome, including retinol metabolism, biogenic amines, amino acids, peptides, glutathione metabolism, fatty acids, and lipid metabolites (4, 6, 12). There are also close connections between changes in the (cervico)vaginal microbiome and metabolome during HPV infection (4, 6, 12). Furthermore, short-chain fatty acids (SCFAs) have been shown to inhibit the entry and replication of several viruses, including hepatitis B virus, severe acute respiratory syndrome coronavirus 2, porcine epidemic diarrhea virus, equine herpesvirus 1, and influenza A virus (22). However, the impact of SCFAs on cervicovaginal HPV status has not been thoroughly explored, and integrating SCFA data with non-targeted metabolomic data may provide deeper insights into HPV infection and CIN development.

In this cross-sectional study, we characterized the profiles and signatures of cervicovaginal secretion and cervical tissue microbiota, as well as cervicovaginal metabolites, including non-targeted metabolomics and SCFAs, in healthy HPV-negative controls, HPV-positive controls, and women with CIN. Our aim is to determine whether specific cervicovaginal secretion and cervical tissue microbiota and their metabolites are associated with HPV infection and CIN development. By doing so, we hope to uncover potential indicators that could help restore microbial balance and facilitate HPV clearance, thereby preventing subsequent disease progression.

## MATERIALS AND METHODS

### Sample collection and study design

A total of 94 women, aged between 20 and 50, were recruited from the cervical disease diagnosis and treatment center at Shanghai First Maternity and Infant Hospital. Participants were confirmed through pathological evaluation in the department of pathology, categorizing them into three groups: CIN (*n* = 43; CIN I, *n* = 9; CIN II/III, *n* = 34), HPV-positive controls (NH; *n* = 24), and healthy HPV-negative controls (NC; *n* = 27) during the period from January to August 2024. Individuals who were pregnant, lactating, menstruating, had recently used corticosteroids or antibiotics, had known malignancies or immunosuppressive disorders, or were undergoing immunosuppressive therapy were excluded from participation in the study.

Four samples of cervicovaginal secretions were collected: one for pH measurement and HPV genotyping and three for further analyses of the cervicovaginal microbiome, SCFAs, and non-targeted metabolomics. Cervical tissues were obtained during colposcopy. For CIN, the tissue was examined due to the presence of cervical neoplasia, while patients with CIN I, who had persisted for over a year and a half, were selected for examination. HPV DNA was detected using the Hybribio HPV typing kit (Chaozhou Hybribio Biotechnology Corp., Chaozhou, China), as previously described (23). All samples were stored at –80°C until processed.

### Five-region (5R) 16S rRNA gene sequencing

DNA extraction from frozen samples was carried out utilizing the cetyltrimethylammonium bromide (CTAB) method in conjunction with the DP302-02 kit (TianGen, Beijing, China), following the manufacturer’s guidelines. The sequencing of the 5R 16S rRNA was executed by LC-Bio Technology Co., Ltd. (Hangzhou, China). For the analysis of the samples, a 5R amplification technique was employed, as outlined in the prior study (24). Sequencing of the libraries was performed on the Illumina NovaSeq 6000 platform. The reads were demultiplexed for each sample, subsequently filtered, and aligned to the respective five amplified regions based on the primer sequences. To integrate the read counts across the five regions into a unified profiling outcome, the Short Multiple Regions Framework method was applied, addressing a maximum likelihood problem (25). The updated GreenGenes database (see Supplementary Methods) served as the reference point. This approach also facilitates the quantification of microbial relative abundance using the expectation maximization algorithm. It is essential to note that data analysis reliability may be compromised by factors such as low-quality reads and primer splice sequences. Consequently, a series of filtration steps were implemented to identify and eliminate contamination, as detailed previously (24).

We employed QIIME 1 (version 1.8.0) alongside the vegan package (version 2.6.2) to evaluate bacterial diversity. For alpha diversity, we concentrated on key indices, including Chao1, observed species, Shannon, and Simpson. Beta diversity analysis was conducted using permutational multivariate analysis of variance (PERMANOVA), based on Bray-Curtis distances. To examine differences in microbial communities between groups, we performed a Mann-Whitney U-test, with a significance threshold set at *P* < 0.05. Furthermore, we executed receiver operating characteristic (ROC) analysis utilizing the pROC package (version 1.18.5), which encompassed plots generated in relation to the number of variables.

### Quantitative analysis of SCFAs

To begin sample preparation, metabolites were precipitated using an 80% methanol-water solution. For derivatization, 1-ethyl-3-(3-dimethylaminopropyl) carbodiimide solution and 3-nitrophenylhydrazine were added to the sample for analysis via liquid chromatography coupled with tandem mass spectrometry (LC-MS/MS). The target compounds were separated and quantified utilizing an AB Sciex Jasper ultra-performance liquid chromatograph in conjunction with an AB SCIEX 4500MD triple quadrupole mass spectrometer. Separation was carried out on an Agilent Poroshell 120 EC-C_18_ column (3.0 × 150 mm, 2.7 µm). The mobile phases consisted of (i) pure water and (ii) a 1:1 (vol/vol) mixture of methanol and acetonitrile, with the following parameters: an injection volume of 1 µL and a column temperature of 40°C.

Mass spectrometric analysis was performed using an electrospray ionization Turbo Ion-Spray interface, functioning in both positive and negative ion modes. The ion source parameters were set to a turbo spray temperature of 400°C and an ion spray voltage of –3,000 V (in negative mode). Collision energy for the various multiple reaction monitoring (MRM) transitions was optimized accordingly, and a specified set of MRM transitions was monitored during the analysis. For raw data with missing values, those below the limit of quantification were assigned a value of zero. A Mann-Whitney U-test was conducted on the metabolite measurements between the two sample groups, identifying differential metabolites as having *P* < 0.05. Principal component analysis (PCA) was performed using the “stats” package (v.4.3.2) in R to identify specific differences between the groups.

### Non-targeted metabolomic profiling

Metabolite extraction, LC-MS analysis, and preprocessing of metabolomics data were performed following the methodology outlined in our previous study (26). PCA and partial least square discriminant analysis (PLS-DA) were executed using MetaX, with *P*-values in both PCA and PLS-DA plots calculated via PERMANOVA utilizing Bray-Curtis distance through the R package vegan. The selection of differential metabolites was conducted with a two-sided unpaired *t*-test, applying the criteria for screening as follows: variable importance in projection (VIP) >1, *P* < 0.05, and fold change >1.2. Additional details are provided in the Supplementary Methods.

### Statistical analysis

The classified data were expressed as counts or mean ± standard deviation (SD). Statistical analyses included the χ^2^ test for categorical variables and the two-sided unpaired *t*-test for continuous variables. For comparisons between groups, the two-sided unpaired *t*-test or the nonparametric Mann-Whitney U-test was utilized, depending on the data characteristics. To generate the correlation heatmap between microbiota, metabolites, and clinical indicators, Pearson’s correlation coefficients were calculated using OmicStudio tools available at https://www.omicstudio.cn/tool/62. ROC analysis was performed using the pROC package (version 1.18.5). Network analysis was carried out using Pearson’s correlation method, again employing OmicStudio tools at https://www.omicstudio.cn/tool/64, with a significance level set at *P* < 0.05 and rho >0.2. All statistical analyses were performed using GraphPad Prism 7.0 (GraphPad Software, CA, USA) and SPSS software (standard v.19.0; IBM). *P*-values are indicated as follows: *P < 0.05, **P < 0.01, ***P < 0.001.

## RESULTS

### Participant population

A total of 122 women were recruited for the study. Following HPV genotyping and clinical pathological diagnoses, 94 participants met the eligibility criteria and were divided into three groups: NC, NH, and CIN. The overall characteristics of the participants are detailed in Table 1. When compared to the NC group, the CIN group exhibited significant differences in several indices, including age, body mass index (BMI), cervicovaginal pH, HPV subtypes, and contraceptive methods (*P* < 0.05). The NH group also showed significant differences from the NC group regarding cervicovaginal pH and HPV subtypes (*P* < 0.05). However, no significant differences were observed between the CIN and NH groups.

**TABLE 1:** Baseline clinical characteristics of the women included^*a*^

	NC	NH	CIN	*P*-value^*c*^		
	(*n* = 27)	(*n* = 24)	(*n* = 43)	SIL vs NC	SIL vs NH	NH vs NC
Age (years)	40 ± 7.36	36 ± 7.78	34 ± 7.39	**0.0012**	0.2816	0.0518
BMI (kg/m^2^)	23.28 ± 2.93	22.81 ± 2.68	22.60 ± 2.88	**0.0170**	0.1081	0.4476
Vaginal pH	5.99 ± 0.92	6.95 ± 0.77	7.02 ± 1.18	**0.0003**	0.8000	**0.0002**
HPV, *N* (%)				**0.0000**	0.0838	**0.0000**
High risk	0 (0.0)	18 (75.0)	39 (90.7)			
Medium-low risk	0 (0.0)	6 (25.0)	4 (9.3)			
Not detected	27 (100)	0 (0.0)	0 (0.0)			
Marital status, *N* (%)				0.5220	0.1171	0.0840
Never married	4 (14.8)	9 (37.5)	9 (20.9)			
Married	23 (85.2)	14 (58.3)	34 (79.1)			
Divorced	0 (0.0)	1 (4.2)	0 (0.0)			
Vaginal discharge, *N* (%)				0.0594	0.3106	0.4717
Normal	19 (70.4)	19 (79.2)	38 (88.4)			
Unusual	8 (29.6)	5 (20.8)	5 (11.6)			
Sexual partners, *N* (%)				0.2555	0.2555	1.0000
1–5	27 (100)	27 (100)	41 (95.3)			
>5	0 (0.0)	0 (0.0)	2 (4.7)			
Age at sexual debut, *N* (%)				0.185	0.5245	0.3789
<20	5 (18.5)	4 (16.7)	10 (23.3)			
20–30	20 (74.1)	20 (83.3)	33 (76.7)			
>30	2 (7.4)	0 (0.0)	0 (0.0)			
No. of childbirths, *N* (%)				0.8701	0.5008	0.4554
0	7 (25.9)	5 (20.8)	12 (27.9)			
1	15 (55.6)	17 (70.8)	23 (53.5)			
2	5 (18.5)	2 (8.3)	7 (16.3)			
3	0 (0.0)	0 (0.0)	1 (2.3)			
No. of abortions, *N* (%)				0.4244	0.7882	0.326
0	9 (33.3)	12 (50.0)	21 (48.8)			
1	9 (33.3)	8 (33.3)	12 (27.9)			
2	9 (33.3)	4 (16.7)	10 (23.3)			
Contraception, *N* (%)				**0.0320**	0.4096	0.4769
Nil	9 (33.3)	10 (41.7)	16 (37.2)			
Condoms	11 (40.7)	11 (45.8)	25 (58.1)			
IUCD/IUD/Mirena/Contraceptive^*b*^	7 (25.9)	3 (12.5)	2 (4.7)			


^*a*^ The data are expressed as the number or mean ± SD. *P*-values for categorical and continuous variables were obtained from the chi-square test and *t*-test, respectively.

^*b*^ IUCD, intrauterine contraceptive device; IUD, intrauterine device.

^*c*^ Bold font indicates statistical significance (*p* < 0.05).

**Fig 1:**
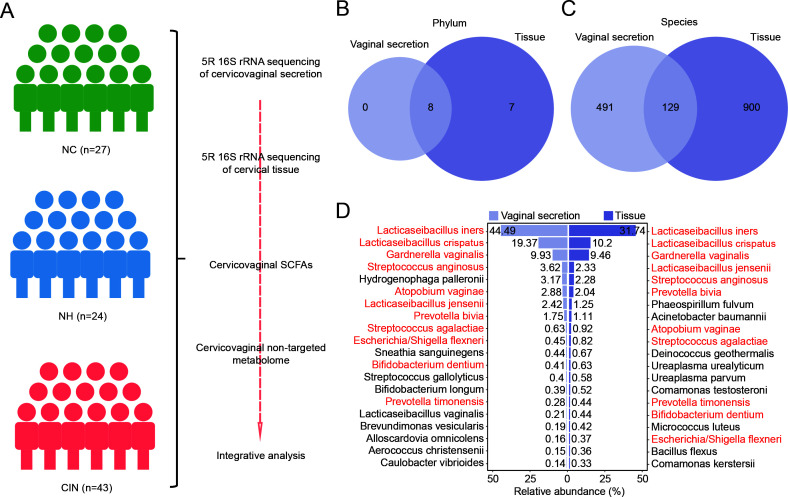
Comparative analysis of the 5R 16S rRNA gene sequencing from cervicovaginal secretion and cervical tissue samples. (**A**) Workflow of the study. Participants underwent 5R 16S rRNA gene sequencing for cervicovaginal secretion and cervical tissue, alongside non-targeted and targeted metabolomic analyses of SCFAs. Data integration was performed for microbiome and metabolome insights. (B–C) Venn diagrams depicting shared and unique microbiota distribution at the phylum (**B**) and species (**C**) levels. (**D**) The butterfly diagram illustrates the top 20 bacterial species identified in cervicovaginal secretion and cervical tissue, emphasizing shared species in red.

For multi-omics analysis, we collected a total of 362 samples from the 94 patients. This included (i) bacterial 5R 16S rRNA gene metabarcoding for 94 cervicovaginal secretion samples; (ii) bacterial 5R 16S rRNA gene metabarcoding for 80 cervical tissue samples; (iii) targeted metabolomic analysis of SCFAs from 94 cervicovaginal secretion samples; and (iv) non-targeted metabolomic analysis from 94 cervicovaginal secretion samples (Fig. 1A).

### Overview the results of the 5R 16S rRNA obtained from cervicovaginal secretion and cervical tissue samples

At the phylum level, we identified eight phyla in cervicovaginal secretions and 15 phyla in cervical tissue samples, with eight phyla common to both (Fig. 1B). At the species level, a total of 620 species were identified in cervicovaginal secretions, while 1,029 species were found in cervical tissue (Fig. 1C). Among these, only 129 species (8.5%) were shared between the two sample types; 491 species (32.3%) were exclusive to cervicovaginal secretions, and 900 species (59.2%) were unique to cervical tissue. Analysis of the 20 most abundant bacterial species showed that 11 species were common across both sample types (Fig. 1D). Notably, dominant species included *Lacticaseibacillus iners*, *Lacticaseibacillus crispatus*, and *Lacticaseibacillus jensenii*, along with anaerobic bacteria such as *Gardnerella vaginalis*, *Atopobium vaginae*, *Streptococcus anginosus*, *Streptococcus agalactiae*, and *Prevotella timonensis*, which align with previous research findings (17, 27). These results indicate that, while there are considerable differences in the microbial compositions of cervicovaginal secretions and cervical tissues, certain key species, particularly *Lacticaseibacillus* and some potential pathogens, play significant ecological roles in both environments.

### Bacterial diversity and composition in cervicovaginal secretions

**Fig 2:**
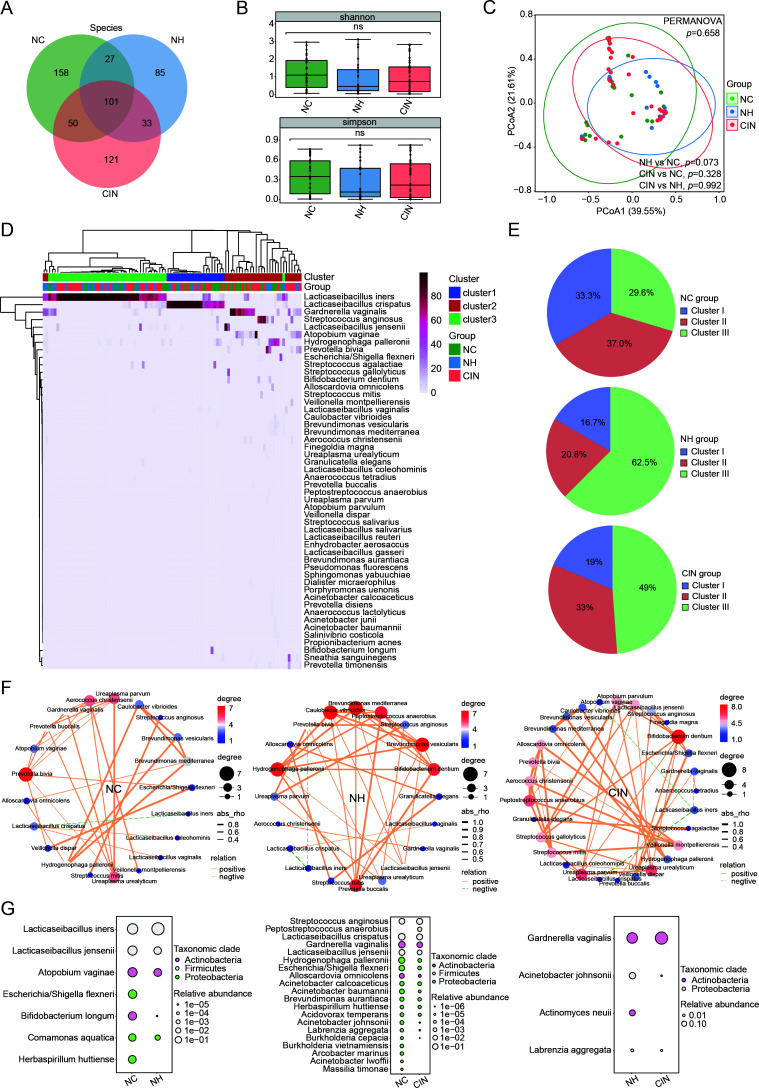
Bacterial diversity and composition analysis of cervicovaginal secretion. (**A**) Venn diagrams comparing bacterial species across NC, NH, and CIN groups. (**B**) Alpha diversity assessed via Shannon (top) and Simpson (bottom) indices. Mann-Whitney U-test was used for *P*-value calculations (between pairs). (**C**) PCoA of beta diversity based on Bray-Curtis distances with significance assessed through PERMANOVA (three groups) and Mann-Whitney U-test (two groups). (**D**) Heatmap of the 50 most abundant bacterial species across 94 samples. (**E**) Visualization of bacterial clusters within analyzed groups. (**F**) Pearson’s correlation analysis of the top 30 bacterial species across groups, displaying only correlations with |rho| > 0.2 and *P* < 0.05. Node size indicates species abundance; line colors reflect positive (orange) and negative (green) correlations. (**G**) Bubble diagrams show differentially abundant bacterial species identified via Mann-Whitney U-test (*P* < 0.05) among group comparisons.

The amplicon-based 5R 16S rRNA gene sequencing method (24) was employed to evaluate the effects of local excisional treatment on the taxonomic composition of cervicovaginal secretions. At the phylum level, most of the identified phyla were common across the NC, NH, and CIN groups (Fig. S1A). At the species level, we identified 336, 246, and 305 bacterial species in the NC, NH, and CIN groups, respectively (Fig. 2A). Among these, 101 species (17.6%) were found in all three groups, while 158 (27.5%), 85 (14.8%), and 121 (21.0%) were unique to the NC, NH, and CIN groups, respectively. Alpha diversity analysis revealed significant differences only between the CIN and NC groups regarding the Observed features and Chao1 indices (Fig. S1B), with no significant variation detected in the Shannon and Simpson indices (Fig. 2B). Additionally, principal coordinates analysis (PCoA) based on Bray-Curtis distance showed no significant differences between the NH vs NC, CIN vs NC, and CIN vs NH groups (analysis of similarities (ANOSIM), *P* > 0.05) (Fig. 2C).

To explore variations in bacterial community types, we categorized the bacteria into three clusters via unsupervised clustering based on species composition and relative abundance (Fig. 2D and Table S1). Clusters I and III were primarily characterized by *L. crispatus* and *L. iners*, respectively, while cluster II showed a reduction in *Lacticaseibacillus* species coupled with a greater diversity of anaerobic or facultative anaerobic bacteria. Notably, *L. iners*, predominant in cluster III and associated with the occurrence of CIN (28, 29), significantly increased following HPV infection and decreased with CIN formation (Fig. 2E). The distribution analysis revealed that the dominant bacteria in the NH and CIN groups exhibited less similarity to the NC group at both the phylum and species levels (Fig. S1C and D). Furthermore, the bacterial species occurrence network displayed increasing complexity from the HC group to the NH and CIN groups, with edge counts of 31, 38, and 52 for the NC, NH, and CIN groups, respectively (Fig. 2F). This indicates a heightened interconnection among bacterial species, attributed to the elevated presence of specific microbial species, particularly in the CIN group.

Subsequent statistical analysis using the Mann-Whitney U-test identified 7 differentially abundant bacterial species (DABs; *P* < 0.05) in the NH group relative to the NC group, 20 DABs in the CIN group against the NC group, and 4 DABs in the CIN group compared to the NH group (Fig. 2G). These DABs are notably linked to HPV acquisition, persistence, and the progression of CIN.

### Bacterial diversity and composition in cervical tissues

**Fig 3:**
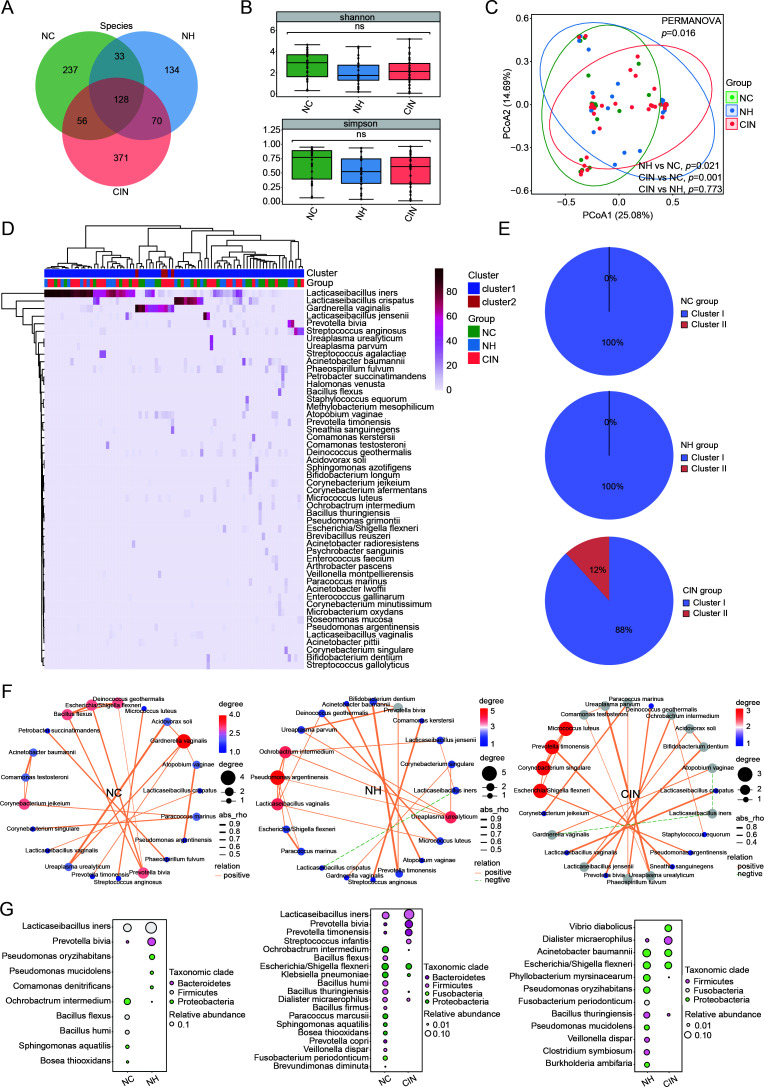
Bacterial diversity and structure analysis of cervical tissue. (**A**) Venn diagrams comparing bacterial species in NC, NH, and CIN women. (**B**) Alpha diversity assessed via Shannon (top) and Simpson (bottom) indices. Mann-Whitney U-test was used for *P*-value calculations (between pairs). (**C**) PCoA of beta diversity based on Bray-Curtis distances with significance assessed through PERMANOVA (three groups) and Mann-Whitney U-test (two groups). (**D**) Heatmap representation of the top 50 bacterial species across 80 cervical tissue samples. (**E**) Cluster visualization for specified groups. (**F**) Pearson’s correlation analysis of the top 30 bacterial species across groups, displaying only correlations with |rho| > 0.2 and *P* < 0.05. Node size indicates species abundance; line colors reflect positive (orange) and negative (green) correlations. (**G**) Bubble diagrams illustrate DABs identified via Mann-Whitney U-test.

To investigate the alterations in taxonomic composition within cervical tissues, we conducted 5R 16S rRNA gene sequencing. Mirroring the patterns observed in cervicovaginal secretions, a significant overlap in phyla was present across the NC, NH, and CIN groups (Fig. S1E). Specifically, 456, 365, and 625 distinct bacterial species were identified in the NC, NH, and CIN groups, respectively (Fig. 3A). Among these, 128 species (12.4%) were common to all three groups, while unique species counts included 237 (23.0%) for NC, 134 (13.0%) for NH, and 370 (36.0%) for CIN. The alpha diversity analysis revealed significant differences in the Observed features index between the NH and NC groups, as well as between the CIN and NC groups, while no notable differences were observed in the Chao1, Shannon, or Simpson indices (Fig. 3B; Fig. S1F). Additionally, PCoA based on Bray-Curtis distance demonstrated significant differences between the NH vs NC (ANOSIM, *P* = 0.021) and CIN vs NC (ANOSIM, *P* = 0.001) groups, while no significant difference was found between CIN and NH groups (ANOSIM, *P* > 0.05) (Fig. 3C).

For a deeper understanding of bacterial community types, we employed unsupervised clustering, which revealed two distinct clusters (Fig. 3D and Table S2). Cluster I comprised primarily *Lacticaseibacillus* species, including *L. iners*, *L. crispatus*, and *L. jensenii*, while cluster II was dominated by *G. vaginalis* and various anaerobic or facultative anaerobic bacteria. Notably, the proportion of cluster II bacteria increased in the CIN group (Fig. 3E). The bacterial classification analysis indicated a less similar distribution of dominant bacteria in the NH and CIN groups compared to the NC group, at both phylum and species levels (Fig. S1G and H). Furthermore, the bacterial species occurrence network revealed similar complexity across all three groups, with edge counts of 20, 23, and 22 for NC, NH, and CIN groups, respectively (Fig. 3F).

Statistical assessments using the Mann-Whitney U-test highlighted 10 DABs (*P* < 0.05) in the NH group relative to the NC group, 19 DABs in the CIN group compared to the NC group, and 12 DABs between the CIN and NH groups (Fig. 3G). Importantly, these DABs may also play a role in HPV acquisition, persistence, and the development of CIN.

### Cervicovaginal metabolomic profiles among NC, NH, and CIN

**Fig 4:**
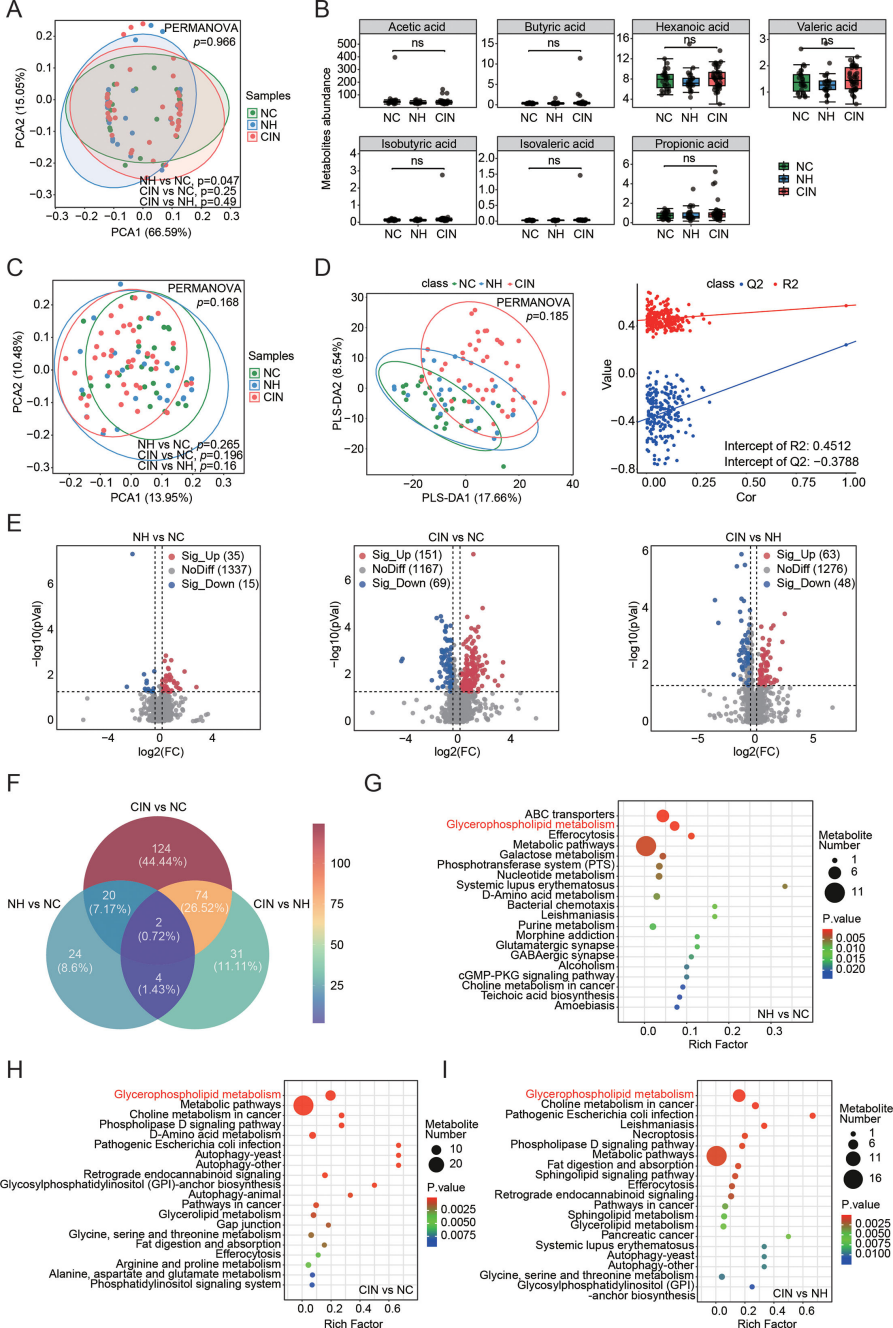
Metabolic profiling of the cervicovaginal metabolome. (**A**) PCA plots showing Bray-Curtis distances for NC, NH, and CIN concerning SCFAs, with *P*-values derived from PERMANOVA for three groups and Mann-Whitney U-test for pairwise group comparisons. (**B**) Box plots depicting SCFA levels among NC, NH, and CIN participants. (**C**) PCA plots utilizing Bray-Curtis distances for NC, NH, and CIN relate to non-targeted metabolomics, with statistical significance assessed through the PERMANOVA test for three groups and the Mann-Whitney U-test for pairwise group comparisons. (**D**) PLS-DA comparison among NC, NH, and CIN, validated with a 200-time permutation test. (**E**) Volcano plots signify metabolic alterations; red dots represent upregulated metabolites, blue for downregulated. Significant metabolites are determined by VIP score (VIP >1, *P* < 0.05, |fold change (FC)| > 1.2). (**F**) Venn diagram illustrating unique and shared differential metabolites across groups. (G–I) Bubble charts detail the top 20 enriched KEGG pathways linked to differential metabolites in each comparison group.

To investigate the metabolomic profiles within the cervicovaginal microenvironment, we utilized a targeted analysis of SCFAs in conjunction with a non-targeted metabolomic approach to assess cervicovaginal excretions. The SCFA profiles showed distinct clustering exclusively between the NH and NC groups, as illustrated by PCA (ANOSIM, *P* = 0.047; Fig. 4A). Subsequent statistical examination using the two-sided unpaired *t*-test indicated no SCFAs were significantly altered between either of the two groups (*P* > 0.05; Fig. 4B).

In the non-targeted metabolomic analysis, a total of 1,387 metabolites were identified. However, the metabolomic profiles revealed no distinct clustering among the NC, NH, and CIN groups, as evidenced by both PCA (PERMANOVA, *P* = 0.168; Fig. 4C) and PLS-DA (Fig. 4D). Further investigations identified 50 metabolites (35 upregulated and 15 downregulated), 220 metabolites (151 upregulated and 69 downregulated), and 111 metabolites (63 upregulated and 48 downregulated) that showed significant alterations in the NH vs NC, CIN vs NC, and CIN vs NH comparisons, respectively (Fig. 4E). Notably, only two metabolites (0.72%) were common across all three groups, while 24 (8.6%), 124 (44.4%), and 31 (11.1%) metabolites were uniquely altered in the NH vs NC, CIN vs NC, and CIN vs NH comparisons, respectively (Fig. 4F). Metabolite enrichment analysis highlighted a significant accumulation of differential metabolites associated with glycerophospholipid metabolism across the groups (Fig. 4G through I; Fig. S2). Given the established roles of lipids in inflammatory responses, these findings suggest their potential contribution to HPV acquisition, persistence, and the development of CIN.

### Pivotal correlations of disrupted cervicovaginal secretion bacteria and metabolites with clinical indices

**Fig 5:**
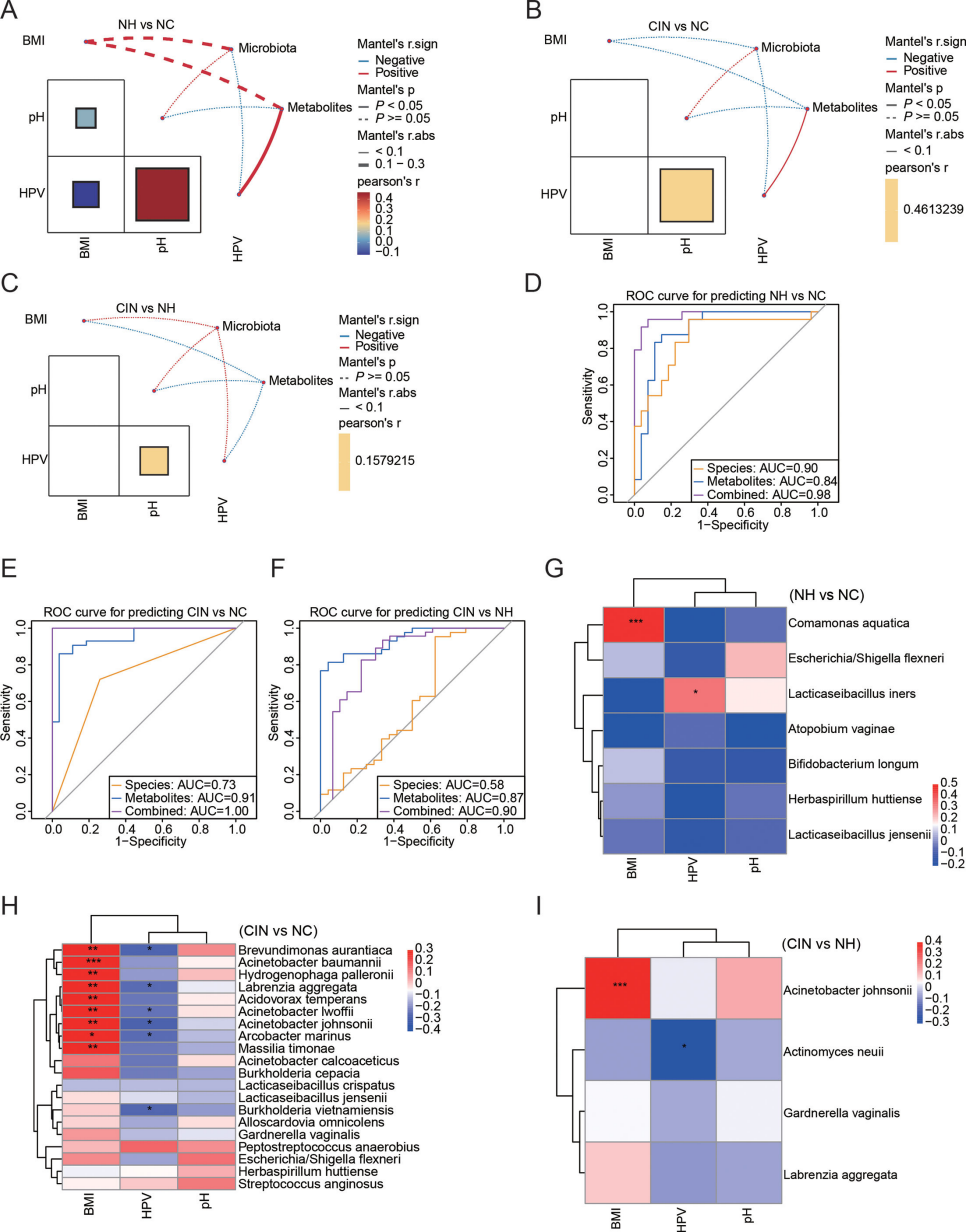
Correlation analysis between altered metabolites, cervicovaginal bacteria, and clinical indices. (A–C) Correlation assessments of clinical indices (BMI, pH, HPV status) against altered metabolites and cervicovaginal DABs. (D–F) ROC curves featuring DABs and metabolites as discriminatory signatures across groups. (G–I) Pearson’s correlation analysis between DABs and clinical indices per group. Significant values are noted as *P < 0.05, **P < 0.01, ***P < 0.001.

To explore the relationships between altered cervicovaginal secretion bacteria and metabolites associated with glycerophospholipid metabolism in relation to clinical indices, we performed Pearson’s correlation analysis. The results revealed significant correlations between entirely altered metabolites and HPV status (*P* < 0.05), particularly in the NH and NC groups, as well as in the CIN and NC groups, which were confirmed by the Mantel test (Fig. 5A through C). We subsequently assessed the potential of these bacterial and metabolomic markers for distinguishing among patient groups. Our predictive model, integrating bacterial species and metabolites, demonstrated high sensitivity in differentiating NH from NC groups (area under the curve [AUC] = 0.98), CIN from NC groups (AUC = 1.0), and CIN and NH groups (AUC = 0.90) (Fig. 5D through F). Furthermore, individual associations among bacteria, metabolites, and clinical indices were examined using Pearson’s correlation analysis (Fig. 5G through I). Notably, we found a positive correlation between *L. iners* and HPV status in the NH and NC groups (Fig. 5G), supported by linear regression analysis (Fig. S3A). Additionally, we identified associations between metabolites, HPV status, and BMI across the comparable groups (Fig. S4), with all interactions further validated through linear regression analysis (Fig. S3B through D). Collectively, these findings indicate a robust interplay between cervicovaginal bacteria, glycerophospholipid metabolism, HPV status, and BMI.

### Pivotal correlations of disrupted cervical tissue bacteria and metabolites with clinical indices

**Fig 6:**
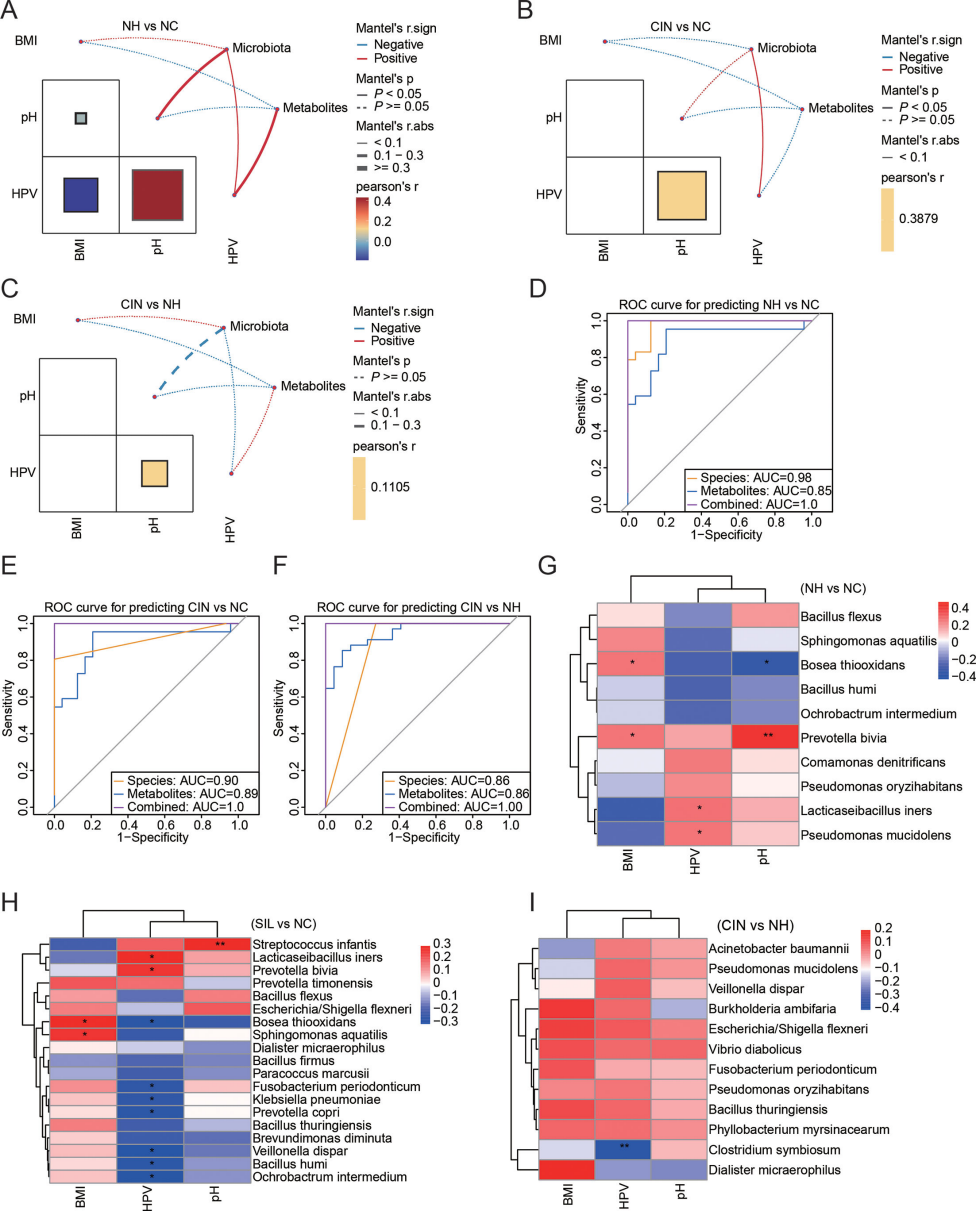
Correlation analysis of altered metabolites, cervical tissue bacteria, and clinical indices. (A–C) Correlation assessments of clinical indices (BMI, pH, HPV status) against altered metabolites and cervical tissue DABs. (D–F) ROC curves featuring DABs and metabolites as discriminatory signatures across groups. (G–I) Pearson’s correlation analysis between DABs and clinical indices per group. Significant values are noted as *P < 0.05, **P < 0.01, ***P < 0.001.

To further investigate the potential connections between altered bacterial communities in cervical tissue and metabolites associated with glycerophospholipid metabolism in relation to clinical indices, we performed Pearson’s correlation analysis. The analysis revealed significant correlations between entirely altered bacteria and metabolites concerning HPV status (NH vs NC; *P* < 0.05), entirely altered bacteria and HPV status (CIN vs NC; *P* < 0.05), as well as entirely altered bacteria and pH changes (NH vs NC; *P* < 0.05), which were supported by the Mantel test (Fig. 6A through C). We then assessed the potential role of these bacterial and metabolomic markers in distinguishing patient groups. Our predictive model demonstrated excellent sensitivity (AUC = 1.0) for differentiating among all three comparison groups (Fig. 6D through F).

To further examine individual correlations between altered bacteria, metabolites, and clinical indices, we again applied Pearson’s correlation analysis (Fig. 6G through I). We identified positive correlations between *L. iners* and HPV, as well as between *P. bivia* and pH in the NH and NC groups, alongside several other positive associations such as *Streptococcus infantis* with pH and *L. iners*, and *P. bivia* with HPV in the CIN and NC groups. Conversely, negative correlations were observed for *Klebsiella pneumoniae*, *Fusobacterium periodonticum*, *Prevotella copri*, and *Veillonella dispar* with HPV in the CIN and NC groups, as well as *Clostridium symbiosum* with HPV from the CIN and NH groups. These relationships were further elucidated through linear regression analysis (Fig. S5A through C). Additionally, we found one positive and six associations (five positive and one negative) linking metabolites with HPV status in the NH and NC groups and CIN and NC groups, respectively (Fig. S6), further investigated by linear regression (Fig. S5D and E). We also noted associations between metabolites and BMI across each group (Fig. S5F and S6). Notably, two specific bacteria-metabolite-HPV linkages—*P*. *bivia*–PE(18:1/0:0)–HPV and *F. periodonticum*–PI 40:6–HPV—emerged from comparisons between CIN and NC groups (Fig. 6H; Fig. S5G, S6B and E). Collectively, these results underscore a significant interplay among cervical tissue bacteria, glycerophospholipid metabolism, HPV status, and BMI.

## DISCUSSION

Our examination of 5R 16S rRNA outcomes from cervicovaginal secretions and cervical tissue samples reveals significant insights into the microbial diversity found within these two environments. The analysis indicates clear compositional variances in the clustering of bacterial communities between the two sample types. At the phylum level, all identified phyla in cervicovaginal samples are present in cervical tissue, suggesting a shared microbial ecosystem that is diverse. However, at the species level, only 8.5% overlap exists between the two environments, hinting that each microenvironment fosters distinct bacterial communities adapted to their unique physiological conditions. Moreover, more than half of the 20 most prevalent bacterial species are common to both environments, highlighting shared ecological interactions. Dominant species include various *Lacticaseibacillus* strains (such as *L. iners*, *L. crispatus*, and *L. jensenii*), as well as potentially pathogenic anaerobes like *G. vaginalis*, *A. vaginae*, alongside different species of *Prevotella* and *Streptococcus*. This finding aligns with previous research (19, 27), emphasizing that while notable differences in microbial composition are present, certain key species—particularly *Lacticaseibacillus* and various anaerobes—are essential in the microbiomes of both cervicovaginal secretions and cervical tissue.

We observed a decrease in the alpha diversity index, specifically in the observed species, between CIN and NC in both cervicovaginal secretions and cervical tissue. However, no significant changes were noted in the Shannon or Simpson indices, which is consistent with the findings of Wu (19) and Nieves-Ramírez et al. (9). This suggests that CIN may contribute to the loss or significant reduction of certain bacterial species. In agreement with previous research (9), we identified alterations in beta diversity within cervical tissue, but not in cervicovaginal secretions, indicating that CIN primarily impacts the tissue microenvironment. Furthermore, the correlation analysis between differential bacteria and HPV status (Fig. S3A, S5A and B) revealed that specific bacteria, such as *L. iners* in both cervicovaginal secretions and cervical tissue, may be involved in mediating HPV infection. Additionally, other bacteria present in cervical tissue, including *P. bivia*, *K. pneumoniae*, *F. periodonticum*, *P. copri*, *V. dispar*, and *C. symbiosum*, may be linked to the persistence of HPV and the progression of CIN.

*L. iners* displays traits of both beneficial lactobacilli under healthy conditions and pathogenic characteristics during dysregulated states (17, 30). It has also been shown to have a strong positive correlation with HPV infection (31). In cervicovaginal secretions, the abundance of *L. iners* initially increases before subsequently decreasing, highlighting its association with HPV infection. Notably, *L. iners* is particularly prevalent in cases of CIN in our study, suggesting its influence on HPV infection and clearance (17). *P. bivia*, an anaerobic bacterium linked to bacterial vaginosis, is known to produce lipopolysaccharides and has the ability to adhere to and invade human cervical epithelial cells, establishing a connection to persistent HPV infection (32, 33). Furthermore, other anaerobic or facultative anaerobic bacteria, such as *K. pneumoniae*, *F. periodonticum*, *P. copri*, and *V. dispar*, are typically commensal but may act as opportunistic pathogens (34–37). A decrease in the abundance of these bacteria can disrupt homeostasis and facilitate the growth of harmful pathogens. These findings suggest that the presence and dynamics of bacteria, such as *L. iners*, *P. bivia*, and other commensals, may contribute to HPV acquisition, persistence, and the progression of CIN.

The current understanding of the metabolic signatures associated with HPV infection and its progression to CIN remains limited. SCFAs, produced by anaerobic bacteria, primarily exert anti-inflammatory effects by inhibiting the expression of adhesion molecules and chemokines, thereby reducing the recruitment of monocytes, macrophages, and neutrophils (38). However, our metabolic profiling showed that there were no significant decreases in SCFA levels in the NH and CIN groups, suggesting that SCFAs may not play a critical role in HPV infection and the development of CIN. Additionally, lipids play a crucial role in the inflammatory process, with alterations in lipid metabolism linked to the progression of various diseases, including CIN (39). Similarly, our analysis also identified glycerophospholipid metabolism as the most significantly dysregulated KEGG pathway in CIN patients compared to NH and NC subjects. Furthermore, the interaction between specific bacteria-metabolites (*P. bivia*–PE(18:1/0:0)–HPV and *F. periodonticum*–PI 40:6–HPV) in CIN and NC groups indicates a significant relationship between cervical tissue bacteria and lipid metabolism, potentially promoting inflammatory responses and facilitating HPV persistence and CIN progression.

Our study presents several strengths, notably its use of multi-omics analysis, which allows for a comprehensive comparison of microbial composition between cervicovaginal secretions and cervical tissues. We employed both targeted and non-targeted metabolomics to characterize the cervicovaginal metabolome, thereby facilitating an investigation into the relationship between metabolite changes and microbiota variations in relation to clinical indicators among healthy women and those infected with HPV. However, the study does have limitations. Firstly, the sample size was relatively small, highlighting the need for further validation in larger and more diverse cohorts from various regions and ethnic backgrounds to support these findings. This limitation also hindered our ability to discern differences based on CIN grades. Secondly, this study is cross-sectional, which limits the ability to establish causal relationships between microbial and metabolic changes and disease progression. Therefore, further longitudinal studies that track these changes over time in HPV-infected individuals are necessary to provide stronger evidence of causality. Thirdly, although the study identifies associations between specific microbial species (e.g., *L. iners* and *P. bivia*) and HPV/CIN, it does not investigate the underlying mechanisms. Future research should examine how these microbes and their metabolites influence HPV persistence and the development of CIN at the molecular level. Lastly, our study did not reveal significant differences in SCFA levels between groups, potentially due to limitations in sample size or variability in detection methods. To address these limitations and further investigate the role of SCFAs in HPV infection and CIN, future research should consider expanding the sample size, incorporating multi-site detection of SCFAs within the local cervical microenvironment, and employing more sensitive detection methods.

In summary, this study identified a variety of bacterial species present in cervicovaginal secretions and cervical tissues, highlighting significant differences in their composition. The analyses demonstrated notable variations in bacterial diversity and composition among the NC, NH, and CIN groups, with specific bacterial species linked to HPV infection and the progression of CIN. For example, *L. iners* was found to be more prevalent in HPV-positive cases but showed a decrease as CIN advanced. Furthermore, the metabolomic analysis revealed alterations associated with glycerophospholipid metabolism; however, no significant changes in SCFAs related to HPV were observed. These metabolites may influence the inflammatory environment, potentially affecting HPV persistence and the development of CIN. The integration of microbiome and metabolomic data effectively differentiated the patient groups, suggesting potential biomarkers for HPV acquisition and CIN progression.

## Data Availability

The 5R 16S rRNA sequencing data for the cervicovaginal secretions reported in this paper have been deposited in Genome Sequence Archive (GSA) with accession CRA022319. The 5R 16S rRNA sequencing data for the cervical tissue reported in this paper have been deposited in GSA with accession CRA022297. The non-targeted metabolomics data reported in this paper have been deposited in OMIX with accession OMIX008731. The targeted metabolomics data for SCFAs reported in this paper have been deposited in OMIX with accession OMIX008718. The authors declare that all the data supporting the findings of this study is available within the paper or from the corresponding authors upon request.

## SUPPLEMENTAL MATERIAL

Supplemental figures spectrum.00192-25-s0001.docx Figures S1 to S6.

Supplemental methods spectrum.00192-25-s0002.docx Non-targeted metabolomic profiling and high-throughput 5R 16S rRNA gene sequencing.

Supplemental tables spectrum.00192-25-s0003.xlsx Tables S1 and S2.

ASM does not own the copyrights to Supplemental Material that may be linked to, or accessed through, an article. The authors have granted ASM a non-exclusive, world-wide license to publish the Supplemental Material files. Please contact the corresponding author directly for reuse.

## References

[1] Mattiuzzi C, Lippi G. 2020. Cancer statistics: a comparison between World Health Organization (WHO) and Global Burden of Disease (GBD). Eur J Public Health 30:1026–1027. doi:10.1093/eurpub/ckz216 31764976

[2] Han B, Zheng R, Zeng H, Wang S, Sun K, Chen R, Li L, Wei W, He J. 2024. Cancer incidence and mortality in China, 2022. J Natl Cancer Cent 4:47–53. doi:10.1016/j.jncc.2024.01.006 39036382 PMC11256708

[3] Perkins RB, Wentzensen N, Guido RS, Schiffman M. 2023. Cervical cancer screening: a review. JAMA 330:547–558. doi:10.1001/jama.2023.13174 37552298

[4] Zhang Y, Wu X, Li D, Huang R, Deng X, Li M, Du F, Zhao Y, Shen J, Chen Y, Zhang P, Hu C, Xiao Z, Wen Q. 2024. HPV-associated cervicovaginal microbiome and host metabolome characteristics. BMC Microbiol 24. doi:10.1186/s12866-024-03244-1 PMC10958955 38519882

[5] Xu H, Liu L, Xu F, Liu M, Song Y, Chen J, Zhan H, Zhang Y, Xu D, Chen Y, Lu M, Chen D. 2022. Microbiome-metabolome analysis reveals cervical lesion alterations. Acta Biochim Biophys Sin (Shanghai) 54:1552–1560. doi:10.3724/abbs.2022149 36269135 PMC9828295

[6] Ilhan ZE, Łaniewski P, Thomas N, Roe DJ, Chase DM, Herbst-Kralovetz MM. 2019. Deciphering the complex interplay between microbiota, HPV, inflammation and cancer through cervicovaginal metabolic profiling. EBioMedicine 44:675–690. doi:10.1016/j.ebiom.2019.04.028 31027917 PMC6604110

[7] Borgogna JC, Shardell MD, Santori EK, Nelson TM, Rath JM, Glover ED, Ravel J, Gravitt PE, Yeoman CJ, Brotman RM. 2020. The vaginal metabolome and microbiota of cervical HPV-positive and HPV-negative women: a cross-sectional analysis. BJOG 127:182–192. doi:10.1111/1471-0528.15981 31749298 PMC6982399

[8] Dong Y-H, Luo Y-H, Liu C-J, Huang W-Y, Feng L, Zou X-Y, Zhou J-Y, Li X-R. 2024. Changes in microbial composition and interaction patterns of female urogenital tract and rectum in response to HPV infection. J Transl Med 22:125. doi:10.1186/s12967-024-04916-2 38303030 PMC10832222

[9] Nieves-Ramírez ME, Partida-Rodríguez O, Moran P, Serrano-Vázquez A, Pérez-Juárez H, Pérez-Rodríguez ME, Arrieta MC, Ximénez-García C, Finlay BB. 2021. Cervical squamous intraepithelial lesions are associated with differences in the vaginal microbiota of mexican women. Microbiol Spectr 9:e0014321. doi:10.1128/Spectrum.00143-21 34643408 PMC8515943

[10] Lin W, Zhang Q, Chen Y, Dong B, Xue H, Lei H, Lu Y, Wei X, Sun P. 2022. Changes of the vaginal microbiota in HPV infection and cervical intraepithelial neoplasia: a cross-sectional analysis. Sci Rep 12:doi doi:10.1038/s41598-022-06731-5 PMC8857277 35181685

[11] Fan Z, Han D, Fan X, Zeng Y, Zhao L. 2024. Analysis of the correlation between cervical HPV infection, cervical lesions and vaginal microecology. Front Cell Infect Microbiol 14:1405789. doi:10.3389/fcimb.2024.1405789 39220285 PMC11362039

[12] Yu T, Gao S, Jin F, Yan B, Wang W, Wang Z. 2024. Characteristics of the vaginal microbiota and vaginal metabolites in women with cervical dysplasia. Front Cell Infect Microbiol 14:1457216. doi:10.3389/fcimb.2024.1457216 39450338 PMC11499233

[13] Mitra A, MacIntyre DA, Lee YS, Smith A, Marchesi JR, Lehne B, Bhatia R, Lyons D, Paraskevaidis E, Li JV, Holmes E, Nicholson JK, Bennett PR, Kyrgiou M. 2015 Cervical intraepithelial neoplasia disease progression is associated with increased vaginal microbiome diversity. Sci Rep 5. doi:10.1038/srep16865 PMC4648063 26574055

[14] Klein C, Gonzalez D, Samwel K, Kahesa C, Mwaiselage J, Aluthge N, et al.. 2019. Relationship between the cervical microbiome HIV status, and precancerous lesions. mBio 10. doi:10.1128/mBio.02785-18 PMC6381280 30782659

[15] Kyrgiou M, Moscicki AB. 2022. Vaginal microbiome and cervical cancer. Semin Cancer Biol 86:189–198. doi:10.1016/j.semcancer.2022.03.005 35276341

[16] Cao Y, Xia H, Tan X, Shi C, Ma Y, Meng D, Zhou M, Lv Z, Wang S, Jin Y. 2024. Intratumoural microbiota: a new frontier in cancer development and therapy. Signal Transduct Target Ther 9:15. doi:10.1038/s41392-023-01693-0 38195689 PMC10776793

[17] Huang R, Liu Z, Sun T, Zhu L. 2024. Cervicovaginal microbiome, high-risk HPV infection and cervical cancer: mechanisms and therapeutic potential. Microbiol Res 287:127857. doi:10.1016/j.micres.2024.127857 39121703

[18] Peng F, Hu M, Su Z, Hu L, Guo L, Yang K. 2024. Intratumoral microbiota as a target for advanced cancer therapeutics. Adv Mater Weinheim 36. doi:10.1002/adma.202405331 39054925

[19] Wu S, Ding X, Kong Y, Acharya S, Wu H, Huang C, Liang Y, Nong X, Chen H. 2021. The feature of cervical microbiota associated with the progression of cervical cancer among reproductive females. Gynecol Oncol 163:348–357. doi:10.1016/j.ygyno.2021.08.016 34503848

[20] Wang H, Jiang Y, Liang Y, Wei L, Zhang W, Li L. 2022. Observation of the cervical microbiome in the progression of cervical intraepithelial neoplasia. BMC Cancer 22:doi doi:10.1186/s12885-022-09452-0 PMC8981842 35379200

[21] Wishart DS, Mandal R, Stanislaus A, Ramirez-Gaona M. 2016. Cancer metabolomics and the human metabolome database. Metabolites 6:10. doi:10.3390/metabo6010010 26950159 PMC4812339

[22] Tsui Y, Wu X, Zhang X, Peng Y, Mok CKP, Chan FKL, Ng SC, Tun HM. 2025. Short-chain fatty acids in viral infection: the underlying mechanisms, opportunities, and challenges. Trends Microbiol 33:302–320. doi:10.1016/j.tim.2024.10.001 39505671

[23] Huang X, Li C, Li F, Zhao J, Wan X, Wang K. 2018. Cervicovaginal microbiota composition correlates with the acquisition of high-risk human papillomavirus types. Int J Cancer 143:621–634. doi:10.1002/ijc.31342 29479697

[24] Nejman D, Livyatan I, Fuks G, Gavert N, Zwang Y, Geller LT, Rotter-Maskowitz A, Weiser R, Mallel G, Gigi E, et al.. 2020. The human tumor microbiome is composed of tumor type-specific intracellular bacteria. Science 368:973–980. doi:10.1126/science.aay9189 32467386 PMC7757858

[25] Fuks G, Elgart M, Amir A, Zeisel A, Turnbaugh PJ, Soen Y, Shental N. 2018. Combining 16S rRNA gene variable regions enables high-resolution microbial community profiling. Microbiome 6:17. doi:10.1186/s40168-017-0396-x 29373999 PMC5787238

[26] Wang J, Pu X, Gu Z. 2024. Clotrimazole-induced shifts in vaginal bacteriome and lipid metabolism: insights into recovery mechanisms in vulvovaginal candidiasis. J Appl Microbiol 135:lxae269. doi:10.1093/jambio/lxae269 39419780

[27] Ravel J, Gajer P, Abdo Z, Schneider GM, Koenig SSK, McCulle SL, Karlebach S, Gorle R, Russell J, Tacket CO, Brotman RM, Davis CC, Ault K, Peralta L, Forney LJ. 2011. Vaginal microbiome of reproductive-age women. Proc Natl Acad Sci USA 108:4680–4687. doi:10.1073/pnas.1002611107 20534435 PMC3063603

[28] Oh HY, Kim B-S, Seo S-S, Kong J-S, Lee J-K, Park S-Y, Hong K-M, Kim H-K, Kim MK. 2015. The association of uterine cervical microbiota with an increased risk for cervical intraepithelial neoplasia in Korea. Clin Microbiol Infect 21:674. doi:10.1016/j.cmi.2015.02.026 25752224

[29] Li C, Zhang Z, Yang Y, Liao H. 2022. Changes in the cervicovaginal microbiota composition of HPV16-infected patients after clinical treatment. Cancer Med 11:5037–5049. doi:10.1002/cam4.4801 35569127 PMC9761074

[30] Petrova MI, Reid G, Vaneechoutte M, Lebeer S. 2017. Lactobacillus iners: friend or foe? Trends Microbiol 25:182–191. doi:10.1016/j.tim.2016.11.007 27914761

[31] Usyk M, Zolnik CP, Castle PE, Porras C, Herrero R, Gradissimo A, Gonzalez P, Safaeian M, Schiffman M, Burk RD, Costa Rica HPV Vaccine Trial (CVT) Group. 2020. Cervicovaginal microbiome and natural history of HPV in a longitudinal study. PLoS Pathog 16:e1008376. doi:10.1371/journal.ppat.1008376 32214382 PMC7098574

[32] Bradshaw CS, Sobel JD. 2016. Current treatment of bacterial vaginosis-limitations and need for innovation. J Infect Dis 214 Suppl 1:S14–20. doi:10.1093/infdis/jiw159 27449869 PMC4957510

[33] Chao X, Sun T, Wang S, Tan X, Fan Q, Shi H, Zhu L, Lang J. 2020. Research of the potential biomarkers in vaginal microbiome for persistent high-risk human papillomavirus infection. Ann Transl Med 8:100. doi:10.21037/atm.2019.12.115 32175393 PMC7049000

[34] Moradigaravand D, Martin V, Peacock SJ, Parkhill J. 2017. Evolution and epidemiology of multidrug-resistant Klebsiella pneumoniae in the United Kingdom and Ireland. MBio 8:doi doi:10.1128/mBio.01976-16 PMC5358916 28223459

[35] Park SN, Park JY, Kook JK. 2010. Development of species-specific polymerase chain reaction primers for detection of Fusobacterium periodonticum. Microbiol Immunol 54:750–753. doi:10.1111/j.1348-0421.2010.00279.x 21223363

[36] Rolhion N, Chassaing B, Nahori M-A, de Bodt J, Moura A, Lecuit M, Dussurget O, Bérard M, Marzorati M, Fehlner-Peach H, Littman DR, Gewirtz AT, Van de Wiele T, Cossart P. 2019. A listeria monocytogenes bacteriocin can target the commensal prevotella copri and modulate intestinal infection. Cell Host & Microbe 26:691–701. doi:10.1016/j.chom.2019.10.016 31726031 PMC6854461

[37] Hung JH, Zhang SM, Huang SL. 2024. Nitrate promotes the growth and the production of short-chain fatty acids and tryptophan from commensal anaerobe Veillonella dispar in the lactate-deficient environment by facilitating the catabolism of glutamate and aspartate. Appl Environ Microbiol 90:e0114824. doi:10.1128/aem.01148-24 39082806 PMC11337843

[38] Vinolo MAR, Rodrigues HG, Nachbar RT, Curi R. 2011. Regulation of inflammation by short chain fatty acids. Nutrients 3:858–876. doi:10.3390/nu3100858 22254083 PMC3257741

[39] Nam M, Seo S-S, Jung S, Jang SY, Lee J, Kwon M, Khan I, Ryu DH, Kim MK, Hwang G-S. 2021. Comparable plasma lipid changes in patients with high-grade cervical intraepithelial neoplasia and patients with cervical cancer. J Proteome Res 20:740–750. doi:10.1021/acs.jproteome.0c00640 33241689

