# Supplementary material for: Microbial and metabolic profiles associated with HPV infection and cervical intraepithelial neoplasia: a multi-omics study
Source: Microbiol Spectr. 2025 Apr 30;13(6):e00192-25. doi: 10.1128/spectrum.00192-25 (PMC12131762; doi:10.1128/spectrum.00192-25)
Supplement: Supplemental figures — Figures S1 to S6. [file spectrum.00192-25-s0001.docx]

**Microbial and metabolic profiles associated with HPV infection and cervical intraepithelial neoplasia: a multi-omics study**

Xiaowen Pu^1,#^, Xiao Wang^1,#^, Jingjing Wang^1^, Zhengrong Gu^1^, Haiyan Zhu^1,*^, Chao Li^2,*^

^1^Department of Gynecology, Shanghai First Maternity and Infant Hospital, School of Medicine, Tongji University, Shanghai 200092, China

^2^Shanghai Key Laboratory of Maternal Fetal Medicine, Shanghai Institute of Maternal-Fetal Medicine and Gynecologic Oncology, Clinical and Translational Research Center, Shanghai First Maternity and Infant Hospital, School of Medicine, Tongji University, Shanghai 200092, China

^#^These authors contributed equally to this work.

^*^**For correspondence:**

Haiyan Zhu, zhuhaiyandoc@sina.com, Department of Gynecology, Shanghai First Maternity and Infant Hospital, School of Medicine, Tongji University, No. 2699 West Gaoke Road, Shanghai 200092, China.

Chao Li, lichao126688@126.com, Shanghai Key Laboratory of Maternal Fetal Medicine, Shanghai Institute of Maternal-Fetal Medicine and Gynecologic Oncology, Clinical and Translational Research Center, Shanghai First Maternity and Infant Hospital, School of Medicine, Tongji University, No. 2699 West Gaoke Road, Shanghai 200092, China.


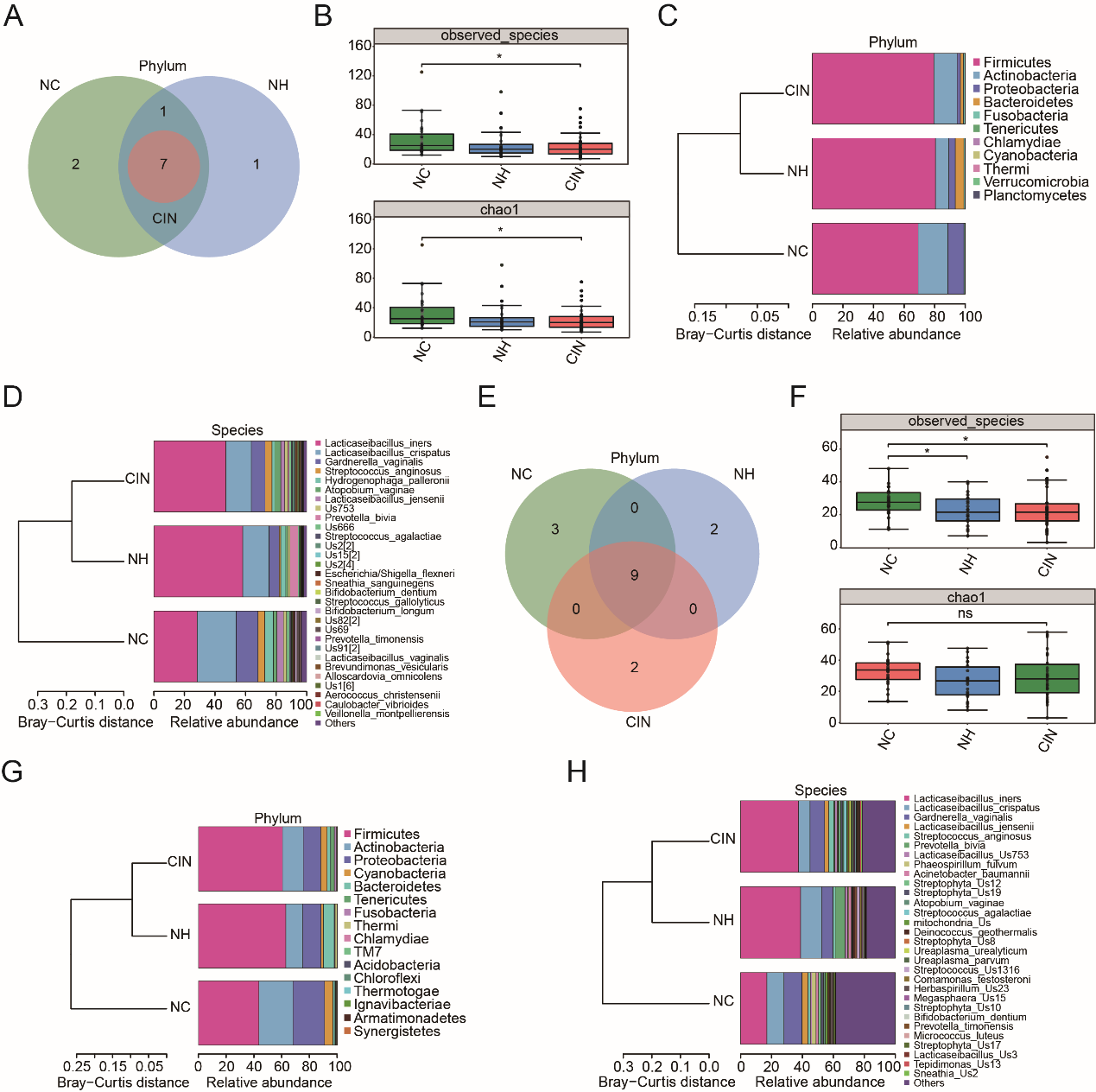


**Figure S1. Overview of 5R 16S rRNA sequencing results from cervicovaginal secretion and cervical tissue.**

(A and E) Venn diagrams show bacterial phyla in cervicovaginal secretion (A) and cervical tissue (E) across NC, NH, and CIN. (B and F) Alpha diversity measures using Observed features and Chao1 indices for cervicovaginal secretion (B) and cervical tissue (F). *P*-values were determined through the Mann-Whitney *U* test.

(C and G) Hierarchical clustering of average phyla proportions from cervicovaginal secretions (C) and cervical tissues (G) among groups based on Bray-Curtis distances.

(D and H) Hierarchical clustering of average species proportions from cervicovaginal secretions (D) and cervical tissues (H) among groups based on Bray-Curtis distances. Us, unknown species.


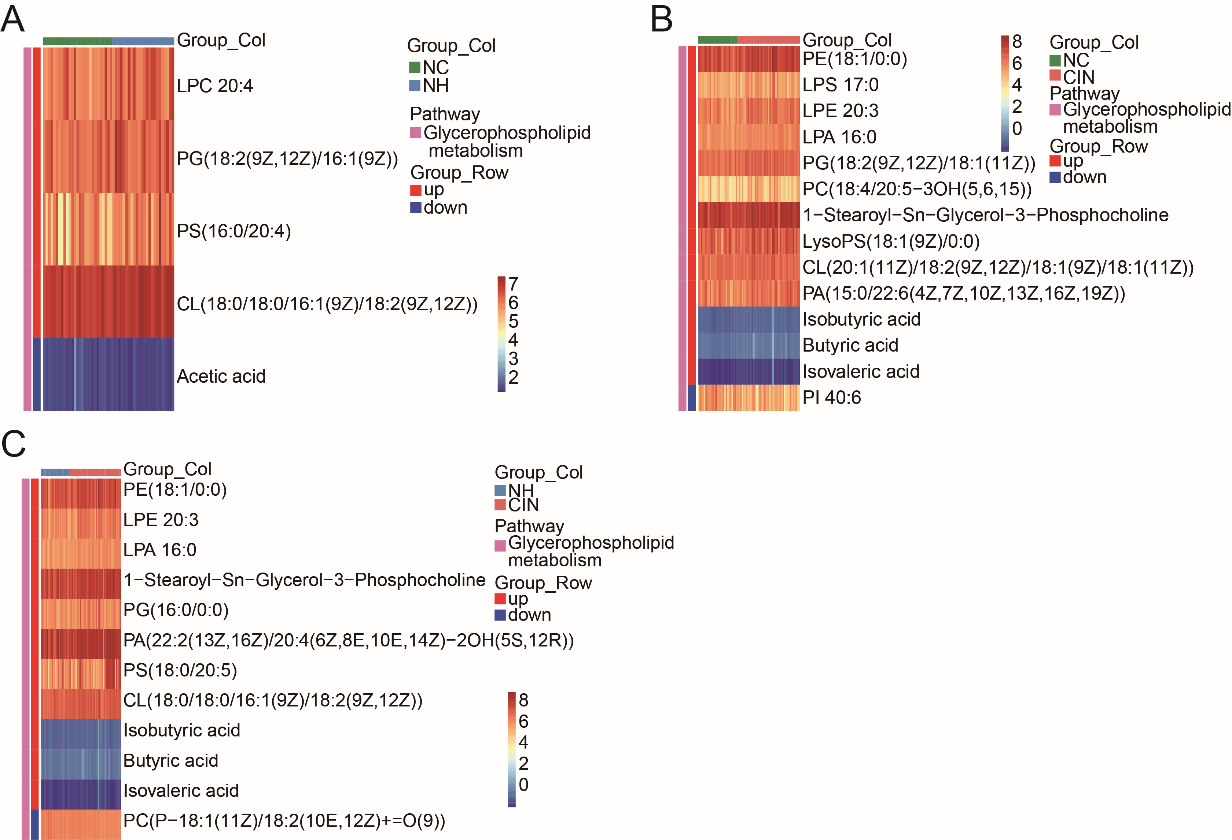


**Figure S2. Heatmaps of significantly altered metabolites related to glycerophospholipid metabolism comparing:** (A) NH vs NC, (B) CIN vs NC, and (C) CIN vs NH.


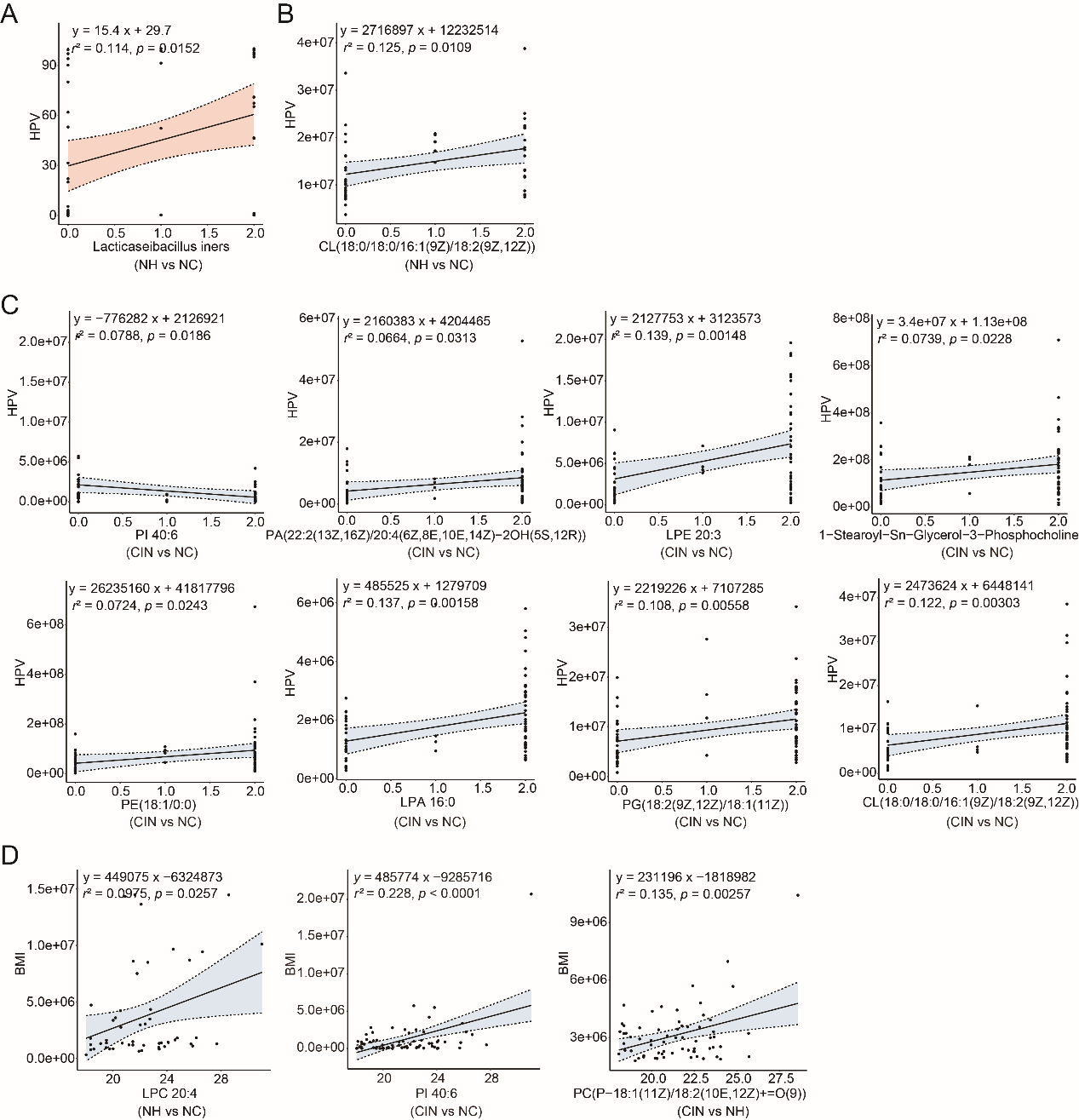


**Figure S3. Correlation analysis among cervicovaginal DABs, altered metabolites, and clinical indices.**

The linear regression analyses reveal the following relationships: (A) the association between *L. iners* and HPV status in NH and NC; (B) the correlation between CL(18:0/18:0/16:1(9Z)/18:2(9Z,12Z)) and HPV status in NH and NC; (C) the link between eight altered metabolites and HPV status in CIN and NC; and (D) the relationship between four altered metabolites and BMI. The solid black line indicates a statistically significant linear relationship (*P* < 0.05), while the shaded regions represent the 95% confidence intervals. Each black dot reflects an individual sample.


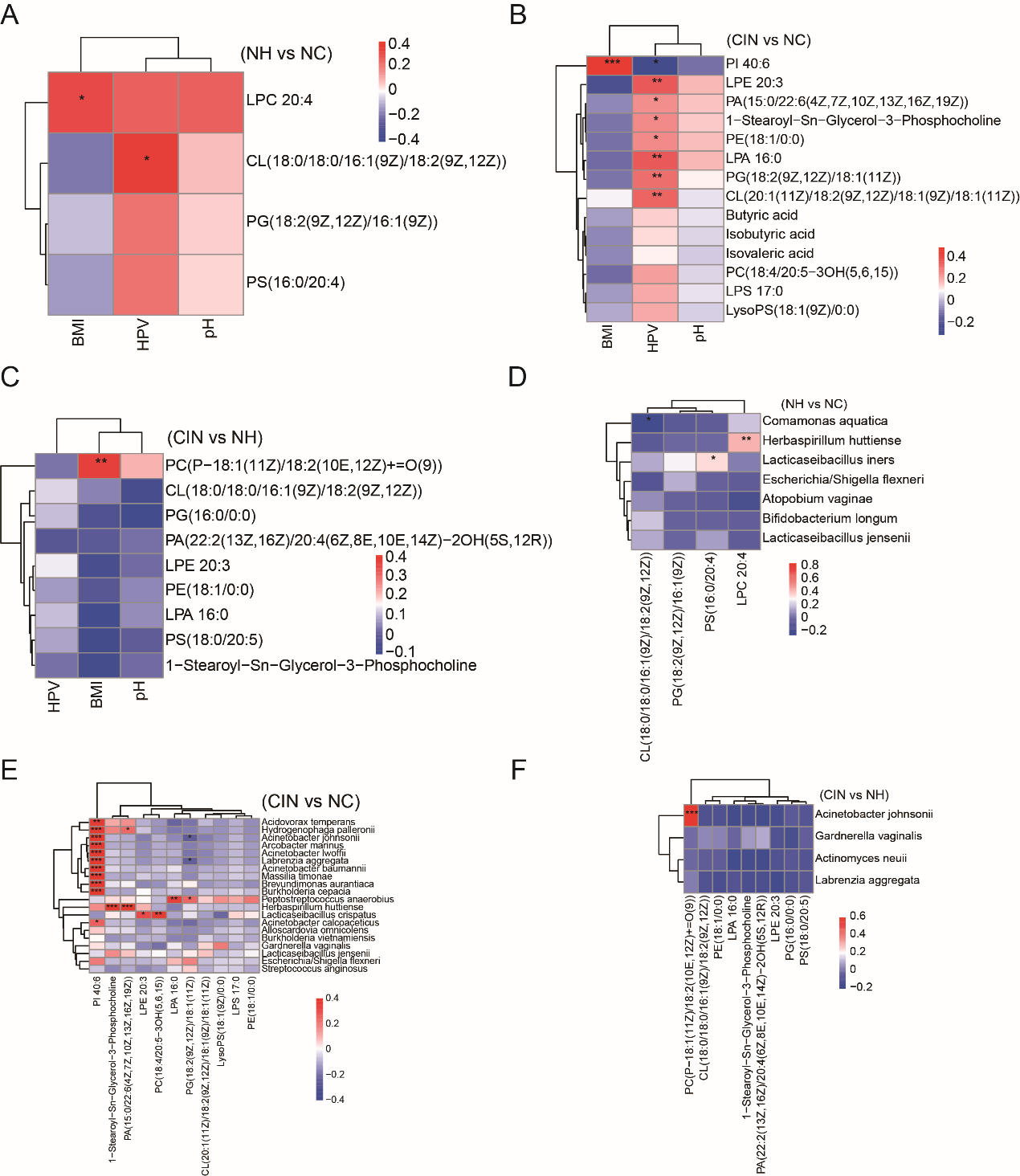


**Figure S4.** Pearson's rank correlation analysis was conducted to examine the relationship between altered metabolites and clinical indices (A–C), as well as between cervicovaginal DABs and altered metabolites (D–F) in each comparison group. Statistical significance was indicated by ^*^*P* < 0.05, ^**^*P* < 0.01, ^***^*P* < 0.001.


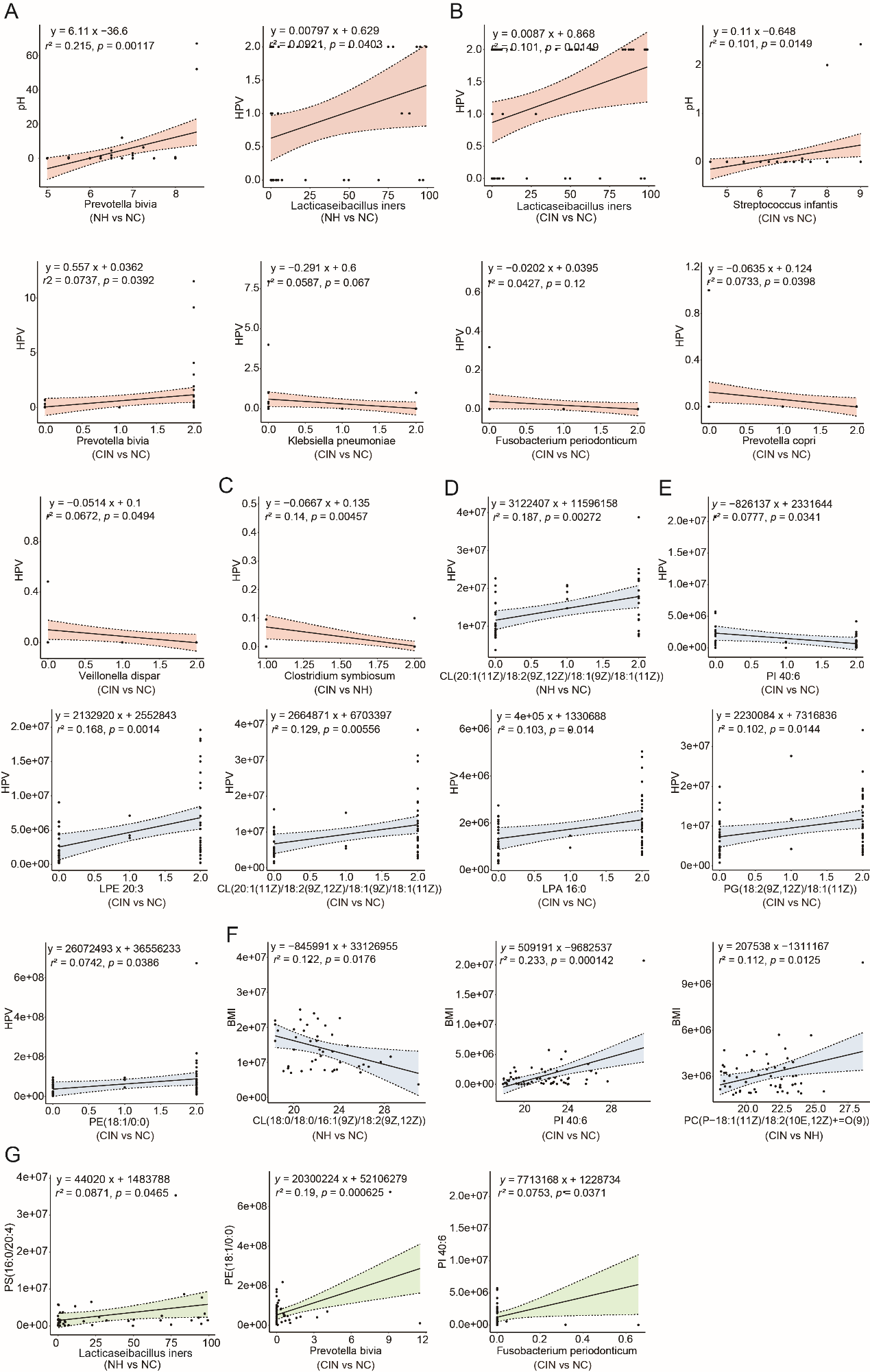


**Figure S5. Correlation assessments among cervical tissue DABs, altered metabolites, and clinical indices.**

The linear regression analyses reveal the following relationships: (A) the correlation between *P. bivia* and pH levels, as well as between *L. iners* and HPV status in NH compared to NC. (B) the association between *S. infantis* and pH values, as well as between *L. iners*, *P. bivia*, *K. pneumoniae*, *F. periodonticum*, *P. copri*, *V. dispar*, and HPV status in CIN compared to NC. (C) the link between *C. symbiosum* and HPV status in CIN compared to NH. (D) the relationship between CL(20:1(11Z)/18:2(9Z,12Z)/18:1(9Z)/18:1(11Z)) and HPV status in NH compared to NC. (E) the connection between the six metabolites and HPV status within CIN compared to NC. (F) the relationship between altered metabolites and BMI. (G) the association between *L. iners* and PS(16:0/20:4), between *P. bivia* and PE(18:1/0:0), or between *F. periodonticum* and PI 40:6 in CIN compared to NC. The solid black line indicates a statistically significant linear association (*P* < 0.05), while shaded areas represent 95% confidence intervals. Each black dot represents an individual sample.


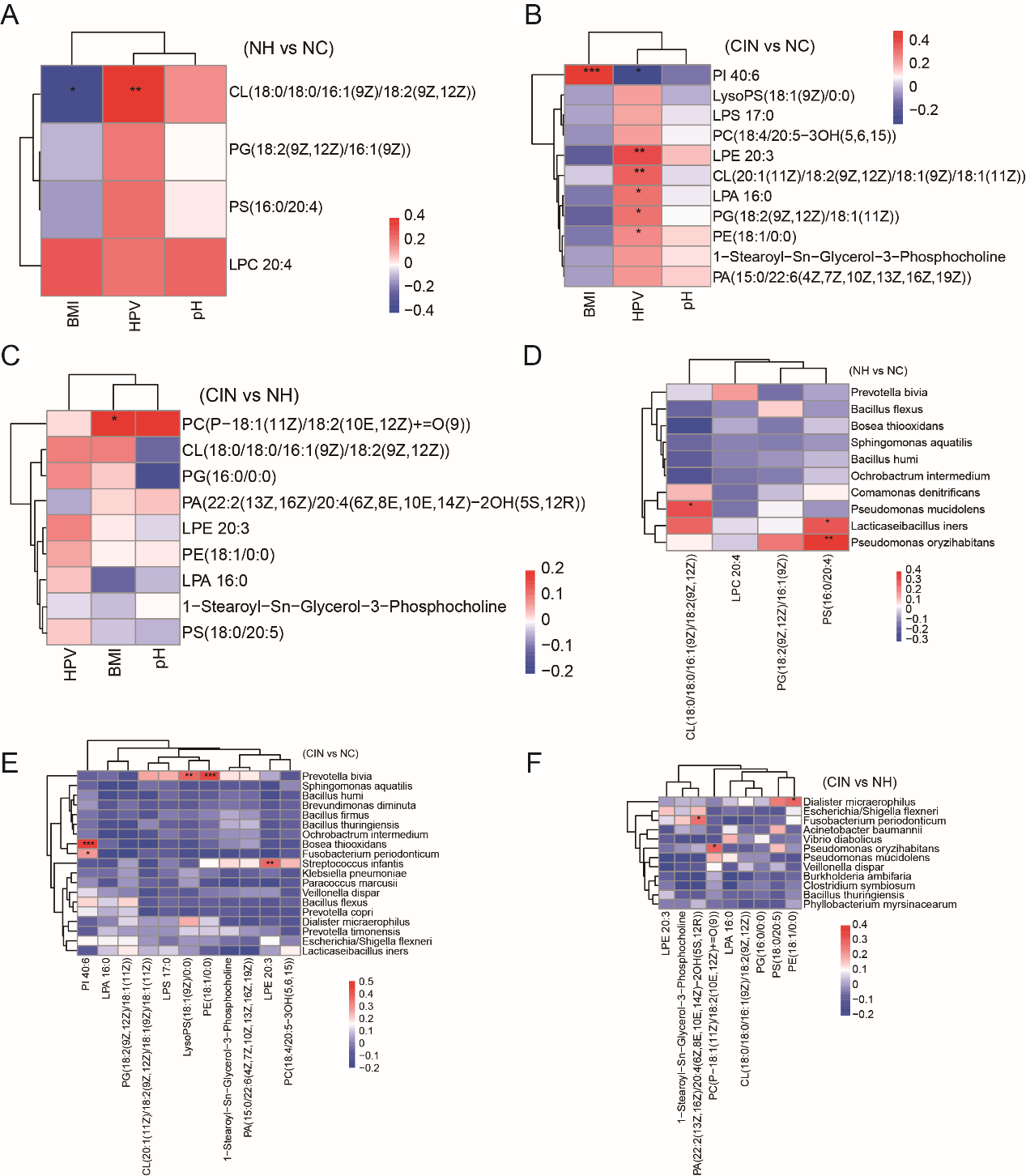


**Figure S6.** Pearson's rank correlation analysis was conducted to examine the relationship between altered metabolites and clinical indices (A–C), as well as between cervical tissue DABs and altered metabolites (D–F) in each comparison group. Statistical significance was indicated by ^*^*P* < 0.05, ^**^*P* < 0.01, ^***^*P* < 0.001.
