# Supplementary material for: Microbial and metabolic profiles associated with HPV infection and cervical intraepithelial neoplasia: a multi-omics study
Source: Microbiol Spectr. 2025 Apr 30;13(6):e00192-25. doi: 10.1128/spectrum.00192-25 (PMC12131762; doi:10.1128/spectrum.00192-25)
Supplement: Supplemental methods — Non-targeted metabolomic profiling and high-throughput 5R 16S rRNA gene sequencing. [file spectrum.00192-25-s0002.docx]

**Microbial and metabolic profiles associated with HPV infection and cervical intraepithelial neoplasia: a multi-omics study**

Xiaowen Pu^1,#^, Xiao Wang^1,#^, Jingjing Wang^1^, Zhengrong Gu^1^, Haiyan Zhu^1,*^, Chao Li^2,*^

^1^Department of Gynecology, Shanghai First Maternity and Infant Hospital, School of Medicine, Tongji University, Shanghai 200092, China

^2^Shanghai Key Laboratory of Maternal Fetal Medicine, Shanghai Institute of Maternal-Fetal Medicine and Gynecologic Oncology, Clinical and Translational Research Center, Shanghai First Maternity and Infant Hospital, School of Medicine, Tongji University, Shanghai 200092, China

^#^These authors contributed equally to this work.

^*^**For correspondence:**

Haiyan Zhu, zhuhaiyandoc@sina.com, Department of Gynecology, Shanghai First Maternity and Infant Hospital, School of Medicine, Tongji University, No. 2699 West Gaoke Road, Shanghai 200092, China.

Chao Li, lichao126688@126.com, Shanghai Key Laboratory of Maternal Fetal Medicine, Shanghai Institute of Maternal-Fetal Medicine and Gynecologic Oncology, Clinical and Translational Research Center, Shanghai First Maternity and Infant Hospital, School of Medicine, Tongji University, No. 2699 West Gaoke Road, Shanghai 200092, China.

**Non-targeted metabolomic profiling**

For metabolite extraction, the collected samples were thawed on ice, and metabolites were extracted using pre-cooled 80% methanol. The mixture was vortexed for 1 minute, incubated for 10 minutes at RT, and then stored at -20°C overnight. After centrifugation at 20,000 × *g* for 15 minutes, the supernatant was transferred to a new EP tube for freeze-drying. The samples were re-dissolved with 100 μL pre-cooled 80% methanol and stored at -80°C until LC-MS analysis. A pooled quality control (QC) sample was prepared by combining 10 μL of each extraction mixture.

For the LC-MS analysis, a TripleTOF 6600 Plus high-resolution tandem mass spectrometer (SCIEX, Warrington, UK) was utilized in both positive and negative ion modes. Chromatographic separation was carried out using an ultra-performance liquid chromatography (UPLC) system (SCIEX, UK) with an ACQUITY UPLC T3 column (100 mm × 2.1 mm, 1.8 µm, Waters, UK) for reversed-phase separation. The mobile phase consisted of solvent A (water, 5 mM ammonium acetate, and 5 mM acetic acid) and solvent B (Acetonitrile). The gradient elution conditions were as follows: 2% solvent B for 0-0.8 min; 2-70% solvent B for 0.8-2.8 min; 70-90% solvent B for 2.8-5.6 min; 90-100% solvent B for 5.6-6.4 min; 100% solvent B for 6.4-8.0 min; 100%-2% solvent B for 8.0-8.1 min; and 2% solvent B for 8.1-10 min. The column temperature was maintained at 40°C. A high-resolution tandem mass spectrometer, TripleTOF 6600 (SCIEX, Framingham, MA, USA), was employed to detect the eluted metabolites. The Q-TOF instrument was operated in both positive and negative ion modes. The curtain gas pressure was maintained at 30 PSI, ion source gas 1 and ion source gas 2 were both set to 60 PSI, and the interface heater temperature was set to 500°C. In positive ion mode, the ion spray voltage floating was adjusted to 5 kV, while in negative ion mode, it was set to -4.5 kV. Mass spectrometry data were acquired using the information-dependent acquisition (IDA) mode, with a mass range from 60 to 1,200 Da. Survey scans were conducted every 150 ms, and up to 12 product ion scans were obtained when exceeding a threshold of 100 counts per second (counts/s) with a 1+ charge state. Dynamic exclusion was implemented for 4 seconds to avoid redundant data collection. Mass accuracy calibration was performed after every 20 samples to ensure precise measurements. To monitor the stability of the LC-MS system throughout the data acquisition process, a quality control sample (comprising a pool of all samples) was analyzed after every 10 samples.

Metabolomics data preprocessing involved a series of steps using XCMS software (v3.4.1) to process the acquired MS data, including peak picking, peak grouping, retention time correction, second peak grouping, and annotation of isotopes and adducts. LC-MS raw data files were initially converted into mzXML format and then processed using the XCMS, CAMERA (v3.4.1), and metaX (v1.4.16) toolbox implemented within the R software (v4.0.0). Identification of each ion was accomplished by combining retention time (RT) and *m/z* data. The intensities of each peak were recorded, resulting in the generation of a three-dimensional matrix containing arbitrarily assigned peak indices (retention time-*m/z* pairs), sample names (observations), and ion intensity information (variables). The online KEGG and HMDB databases were utilized to annotate the metabolites by matching the exact molecular mass data (*m/z*) of samples with those from the database. Metabolites with a mass difference of less than 10 ppm compared to the database value were annotated, and the molecular formulas of metabolites were further identified and validated by isotopic distribution measurements. Additionally, an in-house fragment spectrum library of metabolites was employed to validate the identification of metabolites. Subsequently, the intensity of peak data was further preprocessed by metaX. Features that were detected in less than 50% of QC samples or 80% of biological samples were removed. The remaining peaks with missing values were imputed using the k-nearest neighbor algorithm to enhance the data quality. Principal component analysis (PCA) was then performed to detect outliers and evaluate batch effects using the preprocessed dataset. Quality control-based robust LOESS signal correction was fitted to the QC data with respect to the order of injection to minimize signal intensity drift over time. Furthermore, the relative standard deviations of the metabolic features were calculated across all QC samples, and those exceeding 30% were then removed.

Principal component analysis (PCA) and partial least squares discriminant analysis (PLS-DA) were conducted using MetaX. *P*-values in both PCA and PLS-DA plots were calculated via PERMANOVA based on Bray-Curtis distance using the R package ‘vegan’ to identify differences between groups. Differential metabolites were selected using a Student’s *t*-test, with the following screening criteria: variable importance in projection (VIP) > 1, *P* < 0.05, and fold change > 1.2.

**High-throughput 5R 16S rRNA gene sequencing**

DNA extraction from frozen samples was carried out utilizing the CTAB method in conjunction with the DP302-02 kit (TianGen, Beijing, China), following the manufacturer's guidelines. All negative controls underwent the exact same protocol steps. Different controls were included, such as sampling controls, DNA extraction controls, and no-template controls (NTCs; or PCR controls), to account for the various sources of contamination from the hospital and laboratory environments, as well as the different stages of handling and processing of the samples.

The amplification and sequencing of the 16S rRNA gene were performed as previously described with minor modifications^1^. Specifically, a set of bacterial primers designed to target the V5 region of the 16S rRNA gene were used in the amplification process. Each primer was used at a concentration of 0.1 µM. The forward primers included F1 (TGGCGAACGGGTGAGTAA), F2 (ACTCCTACGGGAGGCAGC), F3 (GTGTAGCGGTGRAATGCG), F4 (GGAGCATGTGGWTTAATTCGA), and F5 (GGAGGAAGGTGGGGATGAC). The reverse primers were R1 (AGACGTGTGCTCTTCCGATCTCCGTGTCTCAGTCCCARTG), R2 (AGACGTGTGCTCTTCCGATCTGTATTACCGCGGCTGCTG), R3 (AGACGTGTGCTCTTCCGATCTCCCGTCAATTCMTTTGAGTT), R4 (AGACGTGTGCTCTTCCGATCTCGTTGCGGGACTTAACCC), and R5 (AGACGTGTGCTCTTCCGATCTAAGGCCCGGGAACGTATT). The amplification process involved using Phusion^®^ Hot Start Flex 2× Master Mix (NEB, #M0536L) along with 50 ng of template DNA in a 25 µl reaction volume. The amplification protocol included an initial heating step of 98˚C for 30 seconds, followed by 30 cycles of denaturation at 98˚C for 10 seconds, annealing at 62˚C for 15 seconds, extension at 72˚C for 35 seconds, and a final elongation step at 72˚C for 5 minutes. Barcodes and Illumina adaptors were incorporated into the amplicon using a secondary PCR reaction involving five forward primers (0.1 µM each primer). The forward primers used were as follows: FF1- AATGATACGGCGACCACCGAGATCTANNNNNNNNTACACTCTTTCCCTACACGACGCTCTTCCGATCTTGGCGAACGGGTGAGTAA, FF2- AATGATACGGCGACCACCGAGATCTANNNNNNNNTACACTCTTTCCCTACACGACGCTCTTCCGATCTACTCCTACGGGAGGCAGC, FF3- AATGATACGGCGACCACCGAGATCTANNNNNNNNTATACACTCTTTCCCTACACGACGCTCTTCCGATCTGTGTAGCGGTGRAATGCG, FF4- AATGATACGGCGACCACCGAGATCTANNNNNNNNTACACTCTTTCCCTACACGACGCTCTTCCGATCTGGAGCATGTGGWTTAATTCGA, FF5- AATGATACGGCGACCACCGAGATCTANNNNNNNNTACACTCTTTCCCTACACGACGCTCTTCCGATCTGGAGGAAGGTGGGGATGAC, alongside one reverse primer (0.1 µM, RR5- CAAGCAGAAGACGGCATACGAGATNNNNNNNNGTGACTGGAGTTCAGACGTGTGCTCTTCCGATCT) incorporating an 8-nucleotide barcode. To increase sequencing throughput and reduce index contamination, unique indices (TA) were incorporated at both P5 and P7 ends. The amplicon, 50 ng, was subjected to further amplification in a 25 µl reaction volume with 6 cycles of denaturation at 98˚C for 10 seconds, annealing at 64˚C for 15 seconds, and extension at 72˚C for 25 seconds. The resulting PCR products were verified using 2% agarose gel electrophoresis. During the DNA extraction process, ultrapure water was used as a negative control instead of a sample solution to avoid false-positive PCR results. The PCR products were purified using AMPure XT beads (Beckman Coulter Genomics, Danvers, MA, USA #A63880) and quantified with Qubit (Invitrogen, USA). The amplicon pools were prepped for sequencing, and both the size and quantity of the amplicon library were assessed using an Agilent 2100 Bioanalyzer (Agilent, USA) and the Library Quantification Kit for Illumina (Kapa Biosciences, Woburn, MA, USA #KK4844). Subsequently, the libraries were sequenced on a NovaSeq 6000 system using a paired-end 2×150 protocol (LC Bio Technology CO., Ltd., Hangzhou, China).

For data quality control, the raw data obtained from sequencing (paired-end data) is subjected to sample data splitting and the statistics of sequencing volume and high-quality base ratio for each sample based on barcode information. The original sequencing data may contain partially low-quality sequences, such as excessively short length, excessive ambiguous bases, and inserted adaptor sequences, etc. To ensure the reliability of subsequent analyses, we meticulously screened and filtered the raw sequence data: 1) Use Cutadapt (v1.9) to identify potential 3' end adaptor sequences (in very few cases, sequence loss may occur) and truncate at the identified adaptor sequences; 2) After removing the 3' end adaptor sequences, use fqtrim (v0.94) to perform quality screening on the sequences using a sliding window method: scan window size is 10 bp, starting from the first base position at the 5' end, and requiring the average base quality in the window to be ≥ Q20 (i.e., average sequencing accuracy of bases > 99%), truncating the sequence at the 3' end base of the first window with an average quality value lower than Q20; 3) Removing sequences with a length less than 100 bp after truncation; 4) Removing sequences with a content of N (ambiguous bases) exceeding 5% after truncation. After the above processing, an effective sequence set (Clean Data) usable for subsequent analysis is obtained, and the proportion it occupies in the original sequencing data is statistically calculated (Clean%).

For species annotation and relative abundance calculation, we employed the Short MUltiple Regions Framework (SMURF)^2^ analysis pipeline to integrate quality-filtered sequence data (including 5 variable regions V2, V3, V5, V6, V8) for preliminary identification of the microbial communities in the samples and calculation of their relative abundances. SMURF utilizes the Expectation-maximization algorithm to integrate and reconstruct sequences from multiple short amplification regions, referencing the Greengenes database (May 2013 version) established by Nejman et al.^1^, to identify the most probable 16S sequence sets for bacterial taxonomic identification and relative abundance calculation. To mitigate the influence of low abundance noise on subsequent analysis, the sequence read count for each sample was normalized, and samples with a total read count < 1000 (including negative controls) and bacterial data with a relative abundance < 10^-4^ were removed. Subsequently, a strict contaminant filtering process was applied to eliminate common environmental and experimental contaminants^1^ setting a threshold of 30% prevalence to identify contaminants, i.e., bacteria appearing in ≥30% of negative control samples were considered contaminants. The remaining microbial information after removal of these contaminants was considered to represent the microbial communities present in the tissues. Since all the samples were processed at a single center and batch, filters 2 to 6 in Nejman D et al.^1^ were no longer applicable. The pipeline and code files for the generated and utilized data are provided at Zenodo with https://doi.org/10.5281/zenodo.15062761..

For the diversity analysis, we utilized Qiime1 (v1.8.0) to perform Alpha diversity analysis and Beta diversity analysis based on the microbial community abundance table, and then used vegan (v2.6.2) for visualization. Alpha diversity was primarily assessed using indices such as Chao1, Observed species, Shannon, and Simpson to reflect the richness and evenness, and was calculated using the Kruskal-Wallis rank sum test and Mann-Whitney *U* test. Beta diversity was assessed using permutational multivariate analysis of variance (PERMANOVA) based on Bray-Curtis distance, and visually represented by principal coordinates analysis (PCoA) to observe differences in microbial community composition between groups. In addition, the distribution of shared and unique microbiota at the species level was visualized using Venn diagram.

To observe the composition and distribution of the most abundant species (dominant species) in each taxonomic level within various samples (or groups), as well as to compare the microbial composition among different groups, ggplot2 (v3.4.0) was employed to calculate the relative abundance of each bacterial species at each taxonomic level in relation to the total species abundance ratio (i.e., relative abundance) and to construct stacked bar charts representing the distribution of bacterial species in different samples (or groups) at various taxonomic levels. To investigate the differences in microbial communities between groups, we conducted a Mann–Whitney *U* test and visualized the results with a bubble diagram using the OmicStudio tools available at <https://www.omicstudio.cn/tool/30>, setting the significance threshold at *P* < 0.05.

**References**

1. Nejman D, Livyatan I, Fuks G, Gavert N, Zwang Y, Geller LT*, et al.* The human tumor microbiome is composed of tumor type-specific intracellular bacteria. Science (New York, NY) **2020**;368(6494):973-80.

2. Fuks G, Elgart M, Amir A, Zeisel A, Turnbaugh PJ, Soen Y*, et al.* Combining 16S rRNA gene variable regions enables high-resolution microbial community profiling. Microbiome **2018**;6(1):17.
